# Surface-wave-assisted nonreciprocity in spatio-temporally modulated metasurfaces

**DOI:** 10.1038/s41467-020-15273-1

**Published:** 2020-03-19

**Authors:** Andrew E. Cardin, Sinhara R. Silva, Shai R. Vardeny, Willie J. Padilla, Avadh Saxena, Antoinette J. Taylor, Wilton J. M. Kort-Kamp, Hou-Tong Chen, Diego A. R. Dalvit, Abul K. Azad

**Affiliations:** 1 Center for Integrated Nanotechnologies, Los Alamos National Laboratory, Los Alamos, NM 87545 USA; 2 Department of Electrical and Computer Engineering, Duke University, Durham, NC 27708 USA; 3 Theoretical Division, Los Alamos National Laboratory, Los Alamos, NM 87545 USA

**Keywords:** Microwave photonics, Metamaterials

## Abstract

Emerging photonic functionalities are mostly governed by the fundamental principle of Lorentz reciprocity. Lifting the constraints imposed by this principle could circumvent deleterious effects that limit the performance of photonic systems. Most efforts to date have been limited to waveguide platforms. Here, we propose and experimentally demonstrate a spatio-temporally modulated metasurface capable of complete violation of Lorentz reciprocity by reflecting an incident beam into far-field radiation in forward scattering, but into near-field surface waves in reverse scattering. These observations are shown both in nonreciprocal beam steering and nonreciprocal focusing. We also demonstrate nonreciprocal behavior of propagative-only waves in the frequency- and momentum-domains, and simultaneously in both. We develop a generalized Bloch-Floquet theory which offers physical insights into Lorentz nonreciprocity for arbitrary spatial phase gradients, and its predictions are in excellent agreement with experiments. Our work opens exciting opportunities in applications where free-space nonreciprocal wave propagation is desired.

## Abstract (web summary)

Overcoming reciprocity is important for novel functionalities. Here, the authors demonstrate a spatio-temporally modulated metasurface capable of complete violation of Lorentz reciprocity by reflecting an incident beam into far-field radiation in forward scattering, but into near-field surface waves in reverse scattering.

## Introduction

The electromagnetic Lorentz reciprocity theorem^1–4^ states that in a linear, time-independent system with symmetric constitutive optical tensors, the ratio between received and transmitted fields are the same for forward and time-reversed propagation directions. While most electromagnetic and photonic devices operate under this principle, there are circumstances in which reciprocity has undesirable effects, e.g., photovoltaic cells re-emitting absorbed solar energy or antennas listening to their own echo. In order to break reciprocity, any of the conditions assumed by the Lorentz theorem must be violated. The use of magneto-optical media breaks time-inversion symmetry and results in an asymmetric scattering matrix, thereby accomplishing optical isolation^5,6^; however, bulky magnets are required for external bias rendering this conventional approach infeasible for systems integration. Incorporating nonlinear materials and using the electric field self-biasing effect can also result in optical isolation^7–9^, but the degree of nonreciprocity is power-dependent and typically requires significant interaction lengths. Breakdown of Lorentz reciprocity has also been demonstrated using spatio-temporally modulated waveguides^10–12^ and leaky-wave antennas^13,14^. The space-time modulation approach can be particularly attractive when applied in metasurface platforms due to its advantages in reduced size, improved integrability, and attaining surface-wave-assisted nonreciprocal behavior.

**Fig. 1:**
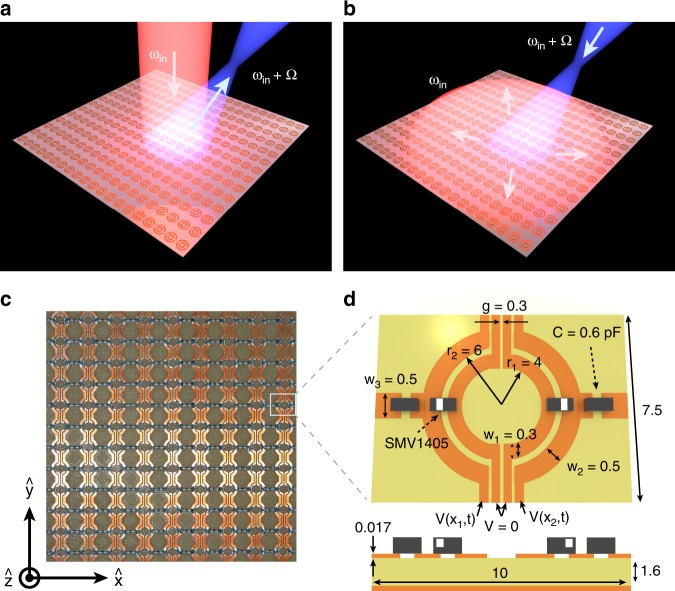
Spatio-temporally modulated metasurfaces. **a** An incident beam impinging on an STMM is converted into a different frequency harmonic that can be focused at any desired focal point. **b** Breakdown of Lorentz reciprocity can be shown by probing the time-reversed process. **c** Photograph of our STMM. **d** Top view and cross-section of the unit cell. All geometrical parameters are in mm.

The advent of metasurfaces^15–20^ has allowed enhanced light-matter interactions within ultra-thin structures, enabling tailored scattering amplitude, phase, and polarization, as well as facilitating the integration of functional materials to accomplish active control^21–25^. Spatially varying phase profiles in judiciously designed static gradient metasurfaces have provided a powerful means to manipulate the momentum harmonic contents of scattered light, ushering in a novel class of flat optics. In addition, active metasurfaces have been shown to be capable of either switching wave-front profiles whenever needed^26–28^, or modulating in time the amplitude and phase of scattered light^29,30^. However, a fully tailored electromagnetic response requires metasurfaces that can continuously alter their scattering properties simultaneously in time and space. These functionalities can be achieved in spatio-temporally modulated metasurfaces^31^ (STMMs), which have the potential to revolutionize fundamental and applied photonics, including nonreciprocity^32,33^, through on-demand control of frequency and momentum harmonic contents of scattered light. For example, an STMM can focus a collimated beam (Fig. 1a), but the time-reversed process is not allowed (Fig. 1b). At microwave frequencies, STMMs can be realized with sub-wavelength metasurface resonators embedded with active elements, such as positive-intrinsic-negative diodes or varactors, which are locally modulated through programmable space-time voltage biases. This concept has been employed for frequency-multiplexed directional scattering within waveguide systems containing time-varying Huygens’ metadevices^34^. For free-space waves, spatio-temporally modulated coding microwave metasurfaces^35,36^ have recently been exploited for nonreciprocal beam steering; however, nonreciprocity limited only to the frequency-domain was accomplished. Also, near-infrared non-linear Kerr metasurfaces^37^ have recently been reported for breakdown of the Lorentz theorem; the setup is limited to frequency-domain nonreciprocal beam steering achieved via a two-beam Bragg configuration, which is difficult to generalize to arbitrary spatio-temporal modulations. A truly multifunctional STMM that can achieve nonreciprocal propagation of arbitrary wave-fronts, and that can even reach photon-to-photon conversion in forward scattering but photon-to-surface wave conversion in reverse scattering has not been experimentally demonstrated to date. In this sense, we call this an “extreme” breakdown of Lorentz reciprocity as the very nature of the photonic modes is modified in the scattering process, and would lead to giant optical isolation as no propagative modes are radiated in reverse scattering.

Here, we present a spatio-temporally modulated metasurface reflectarray capable of dynamically imprinting arbitrary wave-fronts of free-space electromagnetic waves and demonstrate surface-wave-assisted maximum breakdown of the Lorentz theorem through nonreciprocal mode conversion. We exemplify the flexibility of our STMM platform by achieving dynamical beam steering in forward scattering and, when the forward reflection angle is above a certain threshold, we demonstrate nonreciprocity and complete free-space optical isolation by photon-to-surface wave conversion in reverse scattering. Even below threshold, using propagative-only modes we measure nonreciprocal beam steering behavior both in the frequency-domain and momentum-domain, as well as simultaneously in both. In addition, our STMM platform can produce various other kinds of spatial phase distributions, including dynamical focusing. We show nonreciprocal focusing through co-existence of photon-to-surface wave and photon-to-photon excitations in reverse scattering, the former conversion processes overwhelming the latter ones. Importantly, the resulting optical isolation is quasi-optimal and takes place without the need of surpassing any threshold in forward scattering. To model the experimental results, an analytical generalized Bloch-Floquet theory valid for arbitrary spatial phase distributions is developed, which successfully predicts various phenomena measured in the photon-to-surface waves reverse scattering regime. This work can potentially impact emerging technologies benefiting from free-space dynamical wave-front shaping and nonreciprocity, including adaptive optics, Doppler-like frequency translation, echo-immune antennas, and isolated on-chip communications enabled by robust one-way coupling to surface waves.

## Results

### Experimental design

A conceptual illustration of an STMM is shown in Fig. 1a, b, where each unit cell contains active elements that can be independently modulated to impart an arbitrary two-dimensional (2D) phase profile in reflection. In this work, we consider STMMs working at microwave frequencies and varactors as the active elements. Each varactor at position ***r*** on the STMM is subjected to a time-dependent harmonic voltage modulation $V\left( {{\boldsymbol{r}},t} \right)\,=\,V_{{\mathrm{op}}} + \Delta _V\,{\mathrm{sin}}[\varphi ({\boldsymbol{r}}) - {\mathrm{\Omega }}t]$, where *V*_op_ is the operating voltage (or off-set bias), Δ_*V*_ is the voltage modulation amplitude, *φ*(***r***) is the spatial phase distribution, and Ω is the modulation frequency. The voltage modulation translates into a corresponding modulation of the capacitance $C_{{\mathrm{var}}}\left( {{\boldsymbol{r}},t} \right)\,=\,C_{{\mathrm{op}}} + \Delta _C\,{\mathrm{sin}}[\varphi ({\boldsymbol{r}}) - {\mathrm{\Omega }}t]$ as long as a linear relationship between *C*_var_ and *V* holds near *V*_op_.

We design and fabricate an STMM on a commercially available double copper clad FR-4 substrate (Fig. 1c, d). The resonators incorporate two varactor diodes and two capacitors in order to keep a small form factor of the unit cell while providing the required tunability. For simplicity, we restrict the modulation to one dimension by connecting resonators in each column, resulting in spatial modulation among different columns, i.e., along the *x*-direction, but invariant among rows, i.e., *y*-direction (phase delays along this direction can be ignored for Ω ≪ 2π*c*/*L*_*y*_, where *c* is the speed of light in vacuum and *L*_*y*_ is the lateral size of the STMM along *y*). Each column is modulated with a desired voltage and phase using a programmable multi-output function generator. Relative phase shifts among channels can be induced by simultaneous triggering of the system, thereby generating arbitrary digitally constructed waveforms (see Methods and Supplementary Fig. 1).

**Fig. 2:**
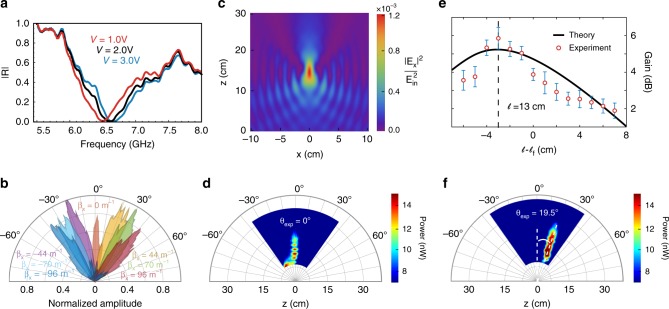
Dynamical wave-front shaping. **a** Reflectance measurements for the unmodulated metasurface. **b** Measured on-demand dynamical beam steering of the *n* = +1 harmonic. **c** Calculated spatial distribution of the electric field for on-axis focusing for the $n = \bar n = + 1$ frequency harmonic (infinite-sized STMM). **d** Measured power for on-axis focusing. **e** Calculated (finite-sized STMM, *L*_*x*_ = 19 cm) and measured gain as a function of $\ell$ for off-axis focusing. Error bars are determined by the standard deviation of gain over a narrow angular range within the focal region. **f** Measured power for off-axis focusing. In **b**–**f**, ***k***_in_ = 0, *ω*_in_ = 2*π* × 6.9 GHz and Ω = 2*π* × 50 kHz. Input power in the experiments is 6.3 mW. Focusing parameters are (*x*_*f*_, *z*_*f*_) = (0,15) cm and (6,15) cm for on-axis and off-axis, respectively.

The reflectivity of the unmodulated metasurface is characterized inside an anechoic chamber at a 10° incidence angle as a function of frequency for various static bias voltages uniformly applied to all varactors. Figure 2a shows measurements for *p*-polarized waves, with the magnetic field component along the *y*-direction. At the operating voltage *V*_op _= 2 V a resonance appears around 6.6 GHz. The optimal working frequency for the modulated STMM, however, is not exclusively determined by the intrinsic resonances of the unmodulated metasurface, but results from an interplay between the unit cell design and the amplitude and phase of the modulation. Indeed, working away from the 6.6 GHz resonance increases the modulation efficiency (see Supplementary Fig. 3c) and thereby the conversion into frequency harmonics (see Supplementary Fig. 4); however, moving too far from the resonance degrades phase dispersion and breaks the linear *C*_var_ vs. *V* relationship (see Supplementary Fig. 3d). For our designed STMM and modulation protocol (Δ_*V*_= 1 V, Ω = 2*π* × 50 kHz), we choose our operational frequency as *ω*_in_ = 2*π* × 6.9 GHz (*λ*_in _= 4.3 cm), which is a good compromise between reflection efficiency, phase dispersion, and linearity.

To test the nonreciprocal functionalities of our spatio-temporally modulated metasurface, we first perform forward scattering measurements by dynamically imprinting appropriate phase delays *φ*(***r***) among the STMM columns. When applying a linear phase delay, we measure the steered beam as a function of scanning angle and frequency in the far-field, while for focusing we apply a parabolic phase profile for a selected frequency harmonic and map the resulting spatial distribution of the scattered power. Subsequently, the corresponding reverse scattering measurements are performed by effectively time-reversing the output wave-fronts of the forward processes, and measuring the far-field scattered power.

### Theoretical formulation

We describe the response of the full array of sub-wavelength resonators with an effective 2D conductivity $\sigma \left( {{\boldsymbol{r}},\omega ;t} \right) = \sigma _{{\mathrm{op}}}\left( \omega \right) + \Delta \sigma \left( \omega \right){\mathrm{sin}}\left[ {\varphi \left( {\boldsymbol{r}} \right) - {\mathrm{\Omega }}t} \right].$ Here, *σ*_op_(*ω*) is the unmodulated complex conductivity at the operating voltage *V*_op_ and Δ*σ*(*ω*) is the complex modulation amplitude. We extract *σ*_op_(*ω*) and estimate Δ*σ*(*ω*) from reflectivity measurements and standard Fresnel equations for multilayered systems (see Supplementary Fig. 3). When an incident plane wave ${\boldsymbol{E}}_\xi ^{{\mathrm{in}}}\left( {{\boldsymbol{r}},z,t} \right) = E^{{\mathrm{in}}} {\hat{\boldsymbol{e}}}_\xi ^ - \left( {{\boldsymbol{k}}_{{\mathrm{in}}}} \right) e^{i({\boldsymbol{r}} \cdot {\boldsymbol{k}}_{{\mathrm{in}}} - z k_{z{\mathrm{v}},{\mathrm{in}}} - \omega _{{\mathrm{in}}}t)}$ with momentum $({\boldsymbol{k}}_{{\mathrm{in}}}, - k_{z{\mathrm{v}},{\mathrm{in}}}{\hat{\mathbf{z}}})$ impinges on the STMM, the reflected field for an arbitrary spatial phase distribution *φ*(***r***) on the STMM is given as

1 $${\boldsymbol{E}}_\xi ^{{\mathrm{refl}}}\left( {{\boldsymbol{r}},z,t} \right) = E^{{\mathrm{in}}}\mathop {\sum}\limits_{n = - \infty }^\infty {{\int} {\frac{{d^2{\boldsymbol{k}}}}{{\left( {2\pi } \right)^2}}} } {\hat{\boldsymbol{e}}}_\xi ^ + \left( {{\boldsymbol{k}}_n} \right)\tilde {\cal{E}}_{\xi ,n}[{\boldsymbol{k}}_{{\mathrm{in}}},{\boldsymbol{k}}_n,\omega _n;{\boldsymbol{r}}]e^{i({\boldsymbol{r}} \cdot {\boldsymbol{k}}_n + zk_{z{\mathrm{v}},n} - \omega _nt)}.$$

Here, ${\hat{\boldsymbol{e}}}_\xi ^ \pm \left( {\boldsymbol{k}} \right)$ are the $\xi = s,p$ polarization unit vectors with ‘+’ and ‘−’ corresponding to waves propagating in the positive and negative *z* directions, respectively; $\omega _n = \omega _{{\mathrm{in}}} + n{\mathrm{\Omega }}$ and ***k***_*n*_ = ***k*** + *n*∇*φ*(***r***) are frequency and momentum harmonics, and $k_{z{\mathrm{v}},n} = \sqrt {(\omega _n/c)^2 - {\boldsymbol{k}}_n^2}$. To compute the reflected field, we develop an analytic Bloch-Floquet approach based on a local derivative expansion of *φ*(***r***), valid for smooth but otherwise general spatial phase distributions. The field amplitude $\tilde {\cal{E}}_{\xi ,n}$ is a functional of both *φ*(***r***) and its gradient ∇*φ*(***r***),

2 $$\tilde {\cal{E}}_{\xi ,n} = \,R_{\xi ,n}\left( {{\boldsymbol{k}},\omega _{{\mathrm{in}}};{\boldsymbol{r}}} \right)\left\{ {\delta _{n,0}\delta ({\boldsymbol{k}} - {\boldsymbol{k}}_{{\mathrm{in}}}) + \frac{{({1 - \delta _{n,0}} ){\mathrm{FT}}[e^{i{\mathrm{sg}}\left( n \right)\varphi \left( {\boldsymbol{r}} \right)}]}}{{e^{i{\mathrm{sg}}\left( n \right)[\varphi \left( {\boldsymbol{r}} \right) - {\boldsymbol{r}} \cdot \nabla \varphi \left( {\boldsymbol{r}} \right)]}}}{\mathrm{\Lambda }}_{\xi ,n}\left[ {{\boldsymbol{k}}_{{\mathrm{in}}},{\boldsymbol{k}}_n,\omega _n;{\boldsymbol{r}}} \right]} \right\}.$$

Here, $R_{\xi ,n}\left( {{\boldsymbol{k}},\omega _{{\mathrm{in}}};{\boldsymbol{r}}} \right)$ is the reflection coefficient corresponding to the scattering process $({\boldsymbol{k}},\omega _{{\mathrm{in}}}) \to \left( {{\boldsymbol{k}}_n,\omega _n} \right)$ at position ***r***, the 2D Fourier transform ${\mathrm{FT}}[e^{i{\mathrm{sg}}(n)\varphi ({\boldsymbol{r}})}]$ is evaluated at ${\boldsymbol{k}} - {\boldsymbol{k}}_{{\mathrm{in}}} + {\mathrm{sg}}(n)\nabla \varphi ({\boldsymbol{r}})$, and ${\mathrm{\Lambda }}_{\xi ,n}$ is defined in the Methods section (see also Supplementary Notes 1–3 for a derivation of the forward scattered fields and the reflection coefficients). Finally, we mention that when the modulation takes place only over a finite-sized region of the metasurface, the reflected field is still given by Eq. (1) after the Fourier transform in Eq. (2) is replaced by ${\mathrm{FT}}[ {\tilde \theta \left( {\boldsymbol{r}} \right)e^{i{\mathrm{sg}}\left( n \right)\varphi \left( {\boldsymbol{r}} \right)}} ]$, where $\tilde \theta \left( {\boldsymbol{r}} \right) = 1$ if ***r*** belongs to the modulated region and is zero otherwise (see Supplementary Note 4 for the generalization of our theory to finite-sized STMMs).

### Dynamical wave-front shaping

In the simplest case of a linear phase distribution $\varphi ^{{\mathrm{steer}}}\left( {\boldsymbol{r}} \right) = {\mathbf{\upbeta }} \cdot {\boldsymbol{r}}$ required for beam steering, the amplitude is $\tilde {\cal{E}}_{\xi ,n}^{{\mathrm{steer}}} = \left( {2\pi } \right)^2R_{\xi ,n}^{{\mathrm{steer}}}\left( {{\boldsymbol{k}},\omega _{{\mathrm{in}}};0} \right)\delta \left( {{\boldsymbol{k}} - {\boldsymbol{k}}_{{\mathrm{in}}}} \right)$. In this case the derivative expansion is exact, and the reflected field is given by a single sum over discrete frequency and momentum harmonics for the scattering processes $({\boldsymbol{k}}_{{\mathrm{in}}},\omega _{{\mathrm{in}}}) \to ({\boldsymbol{k}}_{{\mathrm{in}}} + n{\mathbf{\upbeta }},\omega _{{\mathrm{in}}} + n{\mathrm{\Omega }})$. The azimuthal and polar steering angles are, respectively, given by $\cos \phi _n = {\boldsymbol{k}}_{{\mathrm{in}}} \cdot ({\boldsymbol{k}}_{{\mathrm{in}}} + n{\mathbf{\upbeta }})/\left[ {\left| {{\boldsymbol{k}}_{{\mathrm{in}}}} \right|\left| {{\boldsymbol{k}}_{{\mathrm{in}}} + n{\mathbf{\upbeta }}} \right|} \right]$ and $\sin \theta _n = \left| {{\boldsymbol{k}}_{{\mathrm{in}}} + n{\mathbf{\upbeta }}} \right|c/\omega _n.$ For large enough momentum “kicks” *n****β***, $|\sin \theta _n|$ can become larger than 1 ($k_{z{\mathrm{v}},n}$ purely imaginary) resulting in surface waves, which we later utilize to achieve photon-to-surface waves conversion in reverse scattering. We first demonstrate the multifunctional beam manipulation capability of our STMM by imprinting arbitrary beam steering phase distributions (Fig. 2b). We vary the phase gradient ***β*** along the *x*-direction and show beam steering of the +1 harmonic from +40° to −40°. We observe high quality beam steering with low sidebands and consistent amplitude for a wide range of angles.

To test the flexibility of our STMM for imprinting arbitrary reflection phases, we demonstrate dynamical focusing, a functionality that is relevant, e.g., in compact satellite technologies. The required phase distribution to focus a specific frequency harmonic $\bar n\, \ne\, 0$ at an arbitrary focal point ***R***_*f*_ = (*x*_*f*_, *y*_*f*_, *z*_*f*_) is

3 $$\varphi _{\bar n}^{{\mathrm{focus}}}\left( {{\boldsymbol{k}}_{{\mathrm{in}}},\omega _{{\mathrm{in}}};{\boldsymbol{r}}} \right) = - \frac{1}{{\bar n}}\left\{ {\frac{{\omega _{\bar n}}}{c}\left( {\left| {{\boldsymbol{r}} - {\boldsymbol{R}}_f} \right| - z_f} \right) + {\boldsymbol{k}}_{{\mathrm{in}}} \cdot \left( {{\boldsymbol{r}} - {\boldsymbol{R}}_f} \right)} \right\}.$$

All other harmonics $n \,\ne\, \bar n$ do not result in perfect focusing, and the case *n* = 0 always undergoes specular reflection in STMMs. In contrast to the steering case, the momentum kicks $n\nabla \varphi _{\bar n}^{{\mathrm{focus}}}$ vary as a function of position and the amplitude $\tilde {\cal{E}}_{\xi ,n}^{{\mathrm{focus}}}$ is not proportional to a delta function. These properties hold for any nonlinear phase distribution *φ*_NL_(***r***). Importantly, the phase of the reflected field in Eq. (1) arises from an intricate interplay among the phases of each plane-wave component in the momentum integral. Only for the $n = \bar n$ frequency harmonic does the resulting reflection phase turn out to be precisely given by the focusing distribution Eq. (3) (see Supplementary Note 3 for a discussion of the phase of the total reflection coefficient for the different harmonics). Using our theoretical approach, we first model an infinite-sized STMM subjected to our focusing modulation protocol, that produces a 1D focal line along the *y*-direction, and compute the field distribution for the $n = \bar n = + 1$ harmonic (Fig. 2c).

In Fig. 2d, f we show the experimental results demonstrating the dynamical focusing capability of our STMM (see Methods). Unlike beam steering, which is largely aperture agnostic, focusing requires consideration of the metasurface’s overall dimensions. With a surface in the electric field direction measuring *L*_*x*_ = 19 cm (≈ 4.4 *λ*_in_), a short focal length is necessary for measurable gain, otherwise weak focusing effects are indistinguishable from the usual plane wave like character of the harmonics. For the on-axis focusing case, it is apparent that focusing has been achieved by examination of the increased power and rapid falloff of the signal. At distances $\ell < 12\,{\mathrm{cm}}$ (measured from the STMM center along the focal axis) we observe interference due to near-field coupling between the scanning monopole and the STMM, evident in Fig. 2d. For the off-axis experiment, we measure a much tighter focus due to minimized interference and shadowing effects. To evaluate the quality of the focusing we estimate the gain along the focal axis. We define gain as the ratio between the power of the focused beam at frequency *ω*_+1_ and that of a beam steered at the same frequency *ω*_+1_ into the direction of the focal axis. Figure 2e compares the experimental gain for the off-axis case with the theoretical predictions using the extension of our theory approach to finite-sized STMMs. The experimental data and the theory display the same shape along the focal axis, showing strong agreement. For *x*_*f*_ = 6 cm and *z*_*f*_ = 15 cm, the expected focal length is $\ell _f = 16\,{\mathrm{cm}}$. However, due to the finite size of our metasurface, the gain peaks around $\ell = 13\,{\mathrm{cm}}$, both in experiment and theory (Fig. 2e). At this point, theory predicts a gain of 5.24 dB while the measured value is (5.8 ± 0.6) dB.

### Breakdown of Lorentz reciprocity via near-field surface-waves

**Fig. 3:**
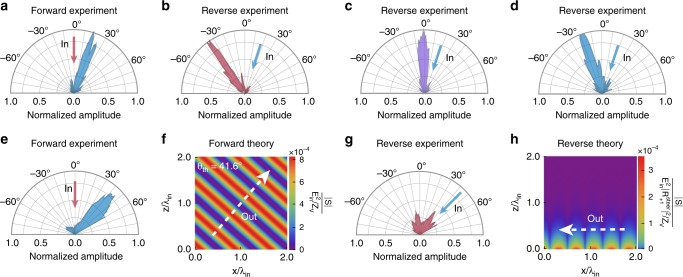
Nonreciprocity in beam steering. **a** A normally-incident (***k***_in _= 0) beam is up-converted to *ω*_in_ + Ω and steered to an angle ≈ +18° with a phase gradient *β*_*x* _= 44 m^−1^. Three dominant reverse pathways are allowed (**b**–**d**). **b** Momentum nonreciprocity: Reflection from +18° of the up-converted beam undergoes a down-conversion to *ω*_in_ and is steered to ≈ −36°. **c** Frequency nonreciprocity: Reflection from +18° undergoes an up-conversion to *ω*_in _+ 2Ω and is normally reflected. **d** Frequency and momentum nonreciprocity: Beam from +18° is specularly reflected at *ω*_in_ + Ω, differing both in frequency and direction of propagation from the original input beam. **e** Forward measured reflection beyond threshold: the input beam is steered to ≈ +43° for *β*_*x* _= 96 m^−1^, which is larger than the +30° threshold. **f** Computed reflected Poynting vector distribution for the forward process in **e**. **g** Measured reverse scattering to *ω*_in_ resulting in photon-to-surface wave conversion and complete optical isolation. **h** Calculated profile of evanescent surface waves corresponding to the reverse process in **g**. In all panels *ω*_in _= 2*π* × 6.9 GHz and Ω = 2*π* × 50 kHz.

We turn our attention to nonreciprocal excitation of surface waves both in beam steering and focusing. In Fig. 3a we report the *n* = +1 beam steered to $\theta _{ + 1} \approx + 18^\circ$ (*β*_*x*_ = 44 m^−1^) from a normally incident plane wave (***k***_in _= 0). In the reverse experiment we send the time-reversed *n* = +1 beam (−***β***, *ω*_in _+ Ω) onto the STMM (see Methods). A frequency down-conversion and a momentum up-conversion must take place in order for the scattering process to be reciprocal. However, this is not permitted due to the traveling-wave nature of the modulation. There exist three dominant reverse reflection pathways: (i) a frequency down-conversion leading to (−***β***, *ω*_in _+ Ω) → (−2***β***, *ω*_in_), clearly breaking reciprocity in the momentum-domain. The measured reverse reflection is shown in Fig. 3b, with a reflection angle ≈ −36° (in contrast to the 0° reciprocal case) in excellent agreement with the theory prediction of −37.5°; (ii) a frequency up-conversion (−***β***, *ω*_in _+ Ω) → (0, *ω*_in _+ 2Ω), resulting in nonreciprocity in the frequency-domain (Fig. 3c); and iii) a specular process (−***β***, *ω*_in_+Ω) → (−***β***, *ω*_in _+ Ω), violating reciprocity both in the frequency-domain and momentum-domain (Fig. 3d), with measured reflection angle ≈ −18°. Finally, we mention that the input wave (***k***_in_, *ω*_in_) can also undergo frequency-conserving specular reflection in the forward experiment, and that in general the backwards specularly reflected field (−***k***_in_, *ω*_in_) satisfies reciprocity. However, under a specific set of parameters it is still possible to attain nonreciprocity in amplitude (not shown). When the magnitude of the phase gradient surpasses a certain threshold ($\left| {\mathbf{\upbeta }} \right| > \omega _{{\mathrm{in}}}/2c = 72.31\,{\mathrm{m}}^{ - 1}$ for ***k***_in _= 0, corresponding to a forward reflection angle of +30°), the reverse scattered beam at frequency ω_in_ does not reflect off the STMM at all. Instead, surface waves are launched on the metasurface as $k_{z{\mathrm{v}}, - 1}^{{\mathrm{REV}},{\mathrm{steer}}} = i\left[ {4|{\mathbf{\upbeta }}|^2 - (\omega _{{\mathrm{in}}}/c)^2} \right]^{1/2}$ is purely imaginary, resulting in photon-to-surface wave conversion, i.e., complete free-space optical isolation. In Fig. 3e, g we present nonreciprocity measurements for *β*_*x*_ = 96 m^−1^ ($\theta _{ + 1} \approx + 43^\circ$), and in Fig. 3f, h the corresponding theory calculations. By scattering an incident beam into far-field collimated radiation in the forward process but into near-field evanescent modes in the reverse case, we demonstrate extreme breakdown of Lorentz reciprocity.

**Fig. 4:**
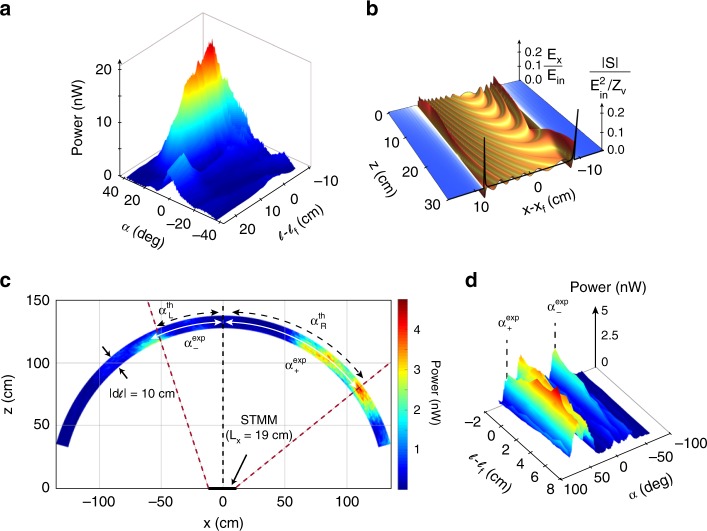
Surface-wave-assisted nonreciprocity in focusing. **a** Measured reflected field power at *ω*_+1_ in the forward off-axis focusing experiment (***k***_in_ = 0). **b** Calculated snapshot of the reflected electric field distribution for the reverse scattering process $( - \nabla \varphi _{\bar n = + 1}^{{\mathrm{focus}}},\omega _{{\mathrm{in}}} + {\mathrm{\Omega }}) \to ( - 2\nabla \varphi _{\bar n = + 1}^{{\mathrm{focus}}},\omega _{{\mathrm{in}}})$ for the infinite-sized STMM. The black line represents the time-averaged Poynting vector for the same scattering process. **c** Measured power at *ω*_in_ in the far-field for the reverse process is mainly confined within the wedge-shaped region. Data at given radii of the arc sector correspond to different locations $\ell$ of the monopole source. Indicated are the calculated main directions of radiative emission from the right and left edges of the STMM and angles subtended between their crossings with the arc and its center, respectively given by $\alpha _{\mathrm{R}}^{{\mathrm{th}}} = + 53.27^\circ$ and $\alpha _{\mathrm{L}}^{{\mathrm{th}}} = - 20.36^\circ$. The measured scanning angles for the main peaks on the right-side and left-side are $\alpha _ + ^{{\mathrm{exp}}} = ( + 54 \pm 1)^\circ$ and $\alpha _ - ^{{\mathrm{exp}}} = ( - 26 \pm 1)^\circ$. **d** Same as **c** but as a function of the scanning angle and $\ell$. Parameters are *x*_*f*_ = 6 cm and *z*_*f*_ = 15 cm $(\ell _f = 16\,{\mathrm{cm}})$. In all panels *ω*_in_ = 2*π* × 6.9 GHz and Ω = 2*π* × 50 kHz.

We now report optical isolation in dynamical focusing. In the forward process a plane wave (0, *ω*_in_) can focus at frequency *ω*_+1_ using the $\varphi _{\bar n = + 1}^{{\mathrm{focus}}}\left( {0,\omega _{{\mathrm{in}}};{\boldsymbol{r}}} \right)$ phase profile. Figure 4a shows the measured power profile of the forward reflected off-axis focused beam as a function of $\ell$ and the angle *α* between the surface normal and the detector. We investigate the scattered field at *ω*_in_ after the focused beam at *ω*_+1_ is time-reversed in order to probe nonreciprocity in the momentum-domain. To grasp some physical insight, we show in Fig. 4b a snapshot of the electric field distribution for the particular scattering process $( - \nabla \varphi _{\bar n = + 1}^{{\mathrm{focus}}},\omega _{{\mathrm{in}}} + {\mathrm{\Omega }}) \to ( - 2\nabla \varphi _{\bar n = + 1}^{{\mathrm{focus}}},\omega _{{\mathrm{in}}})$ for the infinite-sized STMM, similarly to what is done above for nonreciprocal beam steering (see Methods). Also shown is the corresponding time-averaged Poynting vector. The reverse scattered field does not collimate back into the direction of the original input beam, but instead shows a “ribbon-like” region (centered at *x*_*f*_ and of width $2z_f/\sqrt 3$ for the chosen process) containing propagative modes ($k_{z{\mathrm{v}}, - 1}^{{\mathrm{REV}},{\mathrm{focus}}} = \left[ {(\omega _{{\mathrm{in}}}/c)^2 - 4|\nabla \varphi _{\bar n = + 1}^{{\mathrm{focus}}}|^2} \right]^{1/2}$ is real) that radiate away from the metasurface, and is flanked by a region of surface waves with purely imaginary $k_{z{\mathrm{v}}, - 1}^{{\mathrm{REV}},{\mathrm{focus}}}$ (these latter modes move along the STMM and weakly manifest in Fig. 4b due to their strong decay). Other choices of scattering processes also present the same nonreciprocal characteristics. The total reverse scattered field is the sum of all those possible scattering processes and results in maximum breakdown of Lorentz reciprocity (see Supplementary Notes 5 and 6 for further details of Lorentz nonreciprocity for infinite-sized STMM with arbitrary nonlinear spatial phase distributions, including focusing).

For a finite-sized STMM our calculations indicate that a similar physics takes place, the most important difference being that the propagative modes are asymmetrically radiated from the STMM for the off-axis focusing configuration (see Methods and Supplementary Note 7 for a discussion of the Poynting vector in reverse scattering). In Fig. 4c, d we experimentally validate these theoretical predictions by exciting the metasurface with a monopole antenna located at the focal point and emitting at frequency *ω*_+1_, and detect the far-field scattered radiation from the STMM at frequency *ω*_in_. The measured power map bears little resemblance to the original input plane wave. It shows a concave wedge-shaped region with low radiated power at the center, two peaks at asymmetric angular locations, and an outer region of negligible radiated power (displaying similarities to the Poynting vector depicted in Fig. 4b). All these properties of the reverse scattered field are in stark contrast to the input Gaussian profile for the forward process and in excellent agreement with the theory calculations for finite-sized STMMs. In particular, the differences in the measured angular positions of the peaks and in their strengths are due to the inherent asymmetry of off-axis focusing in finite-sized STMMs (see Supplementary Note 7 for a discussion of photon-to-hybrid modes (surface waves and photons) conversion in reverse scattering for finite-sized STMMs for focusing). For positive scanning angles, there is a main peak located at $\alpha _ + ^{{\mathrm{exp}}} = \left( { + 54 \pm 1} \right)^\circ$, while for negative scanning angles there is a much weaker peak at $\alpha _ - ^{{\mathrm{exp}}} = ( - 26 \pm 1)^\circ$.

An extensive discussion of how the STMM emits radiation in reverse scattering can be found in the Supplementary Note 7, where we consider the analytical aspects for the radiative power spectrum in reverse scattering for the focusing functionality. Here, we briefly describe the main results. The structures observed in Fig. 4c, d result from the superposition of emitted radiation from every point on the metasurface. For example, the right-edge at *x* = *L*_*x*_/2 emits radiation mainly in the direction shown in Fig. 4c with a predicted angle $\alpha _{\mathrm{R}}^{{\mathrm{th}}} = + 53.27^\circ$, in very close agreement with the measurement. Points on the metasurface between *x*_*f*_ and *L*_*x*_/2 also contribute to that same peak, resulting in the observed broadening. The second peak seen on the right side of Fig. 4c at a measured position $\alpha _{{\mathrm{ER}}2}^{{\mathrm{exp}}} = ( + 36 \pm 1)^\circ$ arises from secondary emissions from points in the range [*x*_*f*_, *L*_*x*_/2] on the STMM. For example, the right-edge of the structure has a secondary emission direction forming a predicted angle $\alpha _{{\mathrm{ER}}2}^{{\mathrm{th}}} = + 34.66^{\mathrm{o}}$, again in very close agreement with observations. On the other hand, the left-edge of the STMM at *x* = −*L*_*x*_/2 emits radiation mainly in the direction shown in Fig. 4c with a predicted angle $\alpha _{\mathrm{L}}^{{\mathrm{th}}} = - 20.36^\circ$, which is slightly off from the measured position of the peak on the left side of Fig. 4c. The reason for this discrepancy is that all points between −*L*_*x*_/2 and *x*_*f*_ contribute to the peak and its broadening, and there is a given point on the metasurface in the [−*L*_*x*_/2, *x*_*f*_] range that emits precisely in the direction of the peak. Also, in the Supplementary Note 7 we depict the structure of the Poynting vector in reverse scattering, showing a complex co-existence of propagative and evanescent features. In contrast to nonreciprocal beam steering, there is no threshold to launch surface waves in nonreciprocal focusing and evanescent modes overwhelm propagative ones. Just as in nonreciprocal steering, this represents a large violation of the Lorentz reciprocity theorem since in forward scattering there is a photon-to-photon conversion but in reverse scattering there is a photon-to-photon/surface wave hybrid conversion. The measurements reported in Fig. 4c, d constitute the first experimental demonstration of extreme nonreciprocal focusing functionality using STMMs.

In contrast to the case of STMMs modulated with a linear phase *φ*_L_(***r***) = ***β*** · ***r***, for which surface waves in reverse scattering are launched only when its gradient surpasses a certain threshold, no such constraint exists on any nonlinear phase *φ*_NL_(***r***) dynamically imprinted on the metasurface. In this sense, extreme breakdown of Lorentz reciprocity and giant optical isolation are robust properties occurring in space-time metasurfaces under arbitrary nonlinear spatial modulations (see Supplementary Note 8 for a discussion of nonreciprocity in arbitrary nonlinear phase distributions).

## Discussion

In summary, we have introduced an STMM platform for complete violation of Lorentz reciprocity by photon-to-photon conversion in forward scattering, but photon-to-surface wave conversion in reverse scattering. This represents an extreme breakdown of Lorentz reciprocity as the very nature of the photonic modes is modified in the scattering process, leading to giant optical isolation as no propagative modes are radiated in reverse scattering. We also showed that our STMM allows beam-steering nonreciprocity in the frequency-domain, momentum-domain, and simultaneously in both. Our approach enables dynamical arbitrary wave-front shaping by offering substantial flexibility in the manipulation of frequency-momentum harmonic contents of free-space electromagnetic waves when compared to standard phase-gradient and nonlinear optical systems. We have also developed an analytical method to model STMMs with arbitrary spatial phase distributions and its predictions are in excellent agreement with experiment, shedding light on the underlying physics of STMMs with complex phase distributions without resorting to full-wave numerical simulations.

Our goal in this paper is to demonstrate complete breakdown of Lorentz reciprocity via mode-conversion, in the sense explained above. As such, we did not attempt to optimize our structure to achieve either large reflectivity or high efficiency for harmonics conversion. As is clear from Figs. 2 and 3, most of the impinging power gets absorbed by the structure, the reflectivity of the fundamental harmonic is about 4%, and the conversion efficiency into the *n* = +1 harmonic is ~ 0.1%. All these features are a direct consequence of the need to achieve a broad phase dispersion in our designed STMM, which forces us to work close to the near-zero reflection dip (Fig. 2a). Despite the low reflectivity and conversion efficiencies in the forward processes, our nonreciprocal beam steering experiment shows that, beyond a certain threshold, the time-reversed propagative output signal in forward scattering gets completely transformed into evanescent surface waves on the STMM in reverse scattering, thereby achieving extreme nonreciprocity in the direction of propagation and changing the very nature of the photonic modes involved. Similarly, our nonreciprocal focusing measurements show a complete violation of the Lorentz theorem. Tailored metasurfaces with high reflectivity while maintaining a broad phase dispersion can be designed and fabricated for practical applications, as the ones discussed below. However, this by itself does not solve the challenge of substantially enhancing conversion efficiencies into up-converted or down-converted frequency harmonics, as most of the reflected power will still reside in the fundamental harmonic. These limitations can be addressed by using non-harmonic asymmetric time-modulation protocols and higher modulation frequencies, opening opportunities for efficiency improvement, momentum-frequency harmonic mode selectivity, and advanced wave-front manipulation. Using these more general modulation protocols one can in principle attain conversion efficiencies larger than 80%, provided that the complex modulations can be implemented by the electronic controller.

Maximum breakdown of Lorentz reciprocity can occur for any kind of linear or nonlinear spatial phase distributions dynamically imprinted on the metasurface, highlighting that extreme nonreciprocity is a generic property of STMMs. Furthermore, by independently addressing each individual resonator, our platform can be extended to achieve fully three-dimensional dynamical wave-front shaping. STMMs based on our proof-of-concept demonstration with improved designs and complex time-modulation protocols will enable a new architecture for compact and flat multifunctional optical components with built-in isolators, reducing stringent size, weight, and power requirements for wireless communications and remote sensing. In addition, such a spatio-temporally modulated platform can be used to compensate for Doppler shifts induced by relative motion in inter-satellite and Earth-to-satellite communications. Extension of STMMs to the THz and IR frequency range is possible by use of alternative materials and active elements, including back-gate modulated graphene-based resonators, and electro-optic and photo-acoustic media. Finally, our demonstration of maximum violation of Lorentz reciprocity for arbitrary wave-fronts supports emerging technologies benefiting from free-space optical isolation, such as nonreciprocal wireless information transmission.

## Methods

### Device fabrication

The metasurface of dimensions *L*_*x*_ = *L*_*y*_ = 19 cm was fabricated using 12″ × 12″ double sided FR-4 circuit boards, with a thickness *h* = 1.6 mm and 1 oz copper. The top side of the board was milled yielding an array of unit cells on one side, and a continuous ground plane separated by the FR-4 spacer. After milling, the varactors (SMV1405) and capacitors (SMD 0.6 pF) were added by reflow soldering and electrical connections were made to the columns for external control. The voltage signals being fed into each column of the STMM were sourced from a high-density signal generator that is installed on a National Instruments PCI eXtensions for Instrumentation (PXIe) chassis. Each waveform was digitally constructed and uploaded to the onboard buffer of the control system. Both the PXIe chassis and the signal generators have synchronized sample clocks, thus preventing relative phase drift in time and enabling the triggering of all channels to within 10 ns. See Supplementary Fig. 1a for a photograph of the fabricated sample with control lines.

### Measurements

The fabricated metasurface was characterized inside an anechoic chamber using a broad-band horn antenna (SAS-571) and a quarter wave monopole antenna (see Supplementary Fig. 1b, d for a schematic of the experimental set-ups for dynamical steering and focusing, and Supplementary Fig. 1c, e for nonreciprocal steering and focusing). A vector network analyzer (VNA-Agilent N5230A) was used for data collection. First, the reflectivity of the unmodulated metasurface was measured at a 10° incidence angle with *p*-polarized light with all varactors uniformly biased (Supplementary Fig. 2). These measurements were carried out with broadband antennas as the source and receiver, and the VNA was used for a conventional S21 measurement with the source and receiver swept across the band of interest (5.5–8 GHz). We then characterized the spatio-temporally modulated metasurface. For these measurements the source (6.3 mW input power) is in continuous wave (CW) operation at 6.9 GHz with a 100 Hz resolution, and the detector was swept in frequency with corresponding 100 Hz resolution to measure the generated frequency harmonics. For beam steering measurements both the source and the detector were broadband horn antennas; however, for the focusing experiments a monopole antenna was used instead of one of the horn antennas. In the forward experiments the source (CW at ω_in_) was placed on-axis with the metasurface normal $(x = 0,y = 0,z = 1.3)$ m, and the receiver was placed on a computer-controlled gimbal which scanned the reflected power in the *x*−*z* plane. In the beam steering case this yielded radiation patterns of the harmonics scanned along a constant radius (Supplementary Fig. 1b); for focusing experiments a large portion of the *x*−*z* plane was scanned yielding a 2D map of forward radiated power in the harmonics (Supplementary Fig. 1d). For the reverse nonreciprocity experiments the detector and source are swapped, and the new source operates in continuous wave at *ω*_+1_ (Supplementary Fig. 1c, e). The detector measures a frequency sweep as explained above, centered this time at *ω*_in_, capturing harmonics generated around this frequency. The receiver is again scanned in angle as in the forward experiment to measure reflected power over the area of interest.

### Theory

The reflected field by the STMM is described by means of a generalized Bloch-Floquet approach based on a derivative expansion of the spatial phase distributions. It is given by Eq. (1), where $\tilde {\cal{E}}_{\xi ,n}$ is defined in Eq. (2) with ${\mathrm{\Lambda }}_{\xi ,n} = [ {R_{\xi ,n - {\mathrm{sg}}\left( n \right)}\left( {{\boldsymbol{k}}_{{\mathrm{in}}},\omega _{{\mathrm{in}}};{\boldsymbol{r}}} \right) \pm \delta _{\left| n \right|,1}} ]/[R_{\xi ,n - {\mathrm{sg}}\left( n \right)}\left( {{\boldsymbol{k}},\omega _{{\mathrm{in}}};{\boldsymbol{r}}} \right) \pm \delta _{\left| n \right|,1}]$. Here, the ‘+’ and ‘−’ signs correspond to *ξ* = *s* and *ξ* = *p* polarizations, respectively. The reflection coefficients $R_{\xi ,n}\left( {{\boldsymbol{k}},\omega _{{\mathrm{in}}};{\boldsymbol{r}}} \right)$ are obtained by using the local derivative expansion in Maxwell equations and solving numerically (or via continued fractions) an infinite set of coupled equations $\eta _{\xi ,n - 1}^ + {\cal{R}}_{\xi ,n - 1} + A_{\xi ,n}{\cal{R}}_{\xi ,n} + \eta _{\xi ,n + 1}^ - {\cal{R}}_{\xi ,n + 1} = 2B_{\xi ,n}\delta _{n,0}.$ For *p*-polarization, ${\cal{R}}_{p,n}\left( {{\boldsymbol{k}},\omega _{{\mathrm{in}}};{\boldsymbol{r}}} \right) = R_{p,n}\left( {{\boldsymbol{k}},\omega _{{\mathrm{in}}};{\boldsymbol{r}}} \right) - \delta _{n,0}$, $\eta _{p,n}^ \pm = \mp i(Z_{\mathrm{s}}/2)(k_{z{\mathrm{v}},n}/k_{{\mathrm{v}},n})\Delta \sigma \left( {\omega _n} \right)e^{ \pm i[\varphi \left( {\boldsymbol{r}} \right) - {\boldsymbol{r}} \cdot \nabla \varphi \left( {\boldsymbol{r}} \right)]}$, $A_{p,n} = Z_{\mathrm{s}}\sigma _{{\mathrm{op}}}\left( {\omega _n} \right)(k_{z{\mathrm{v}},n}/k_{{\mathrm{v}},n}) + i(k_{z{\mathrm{v}},n}k_{{\mathrm{s}},n}/k_{{\mathrm{v}},n}k_{z{\mathrm{s}},n})\cot ( {hk_{z{\mathrm{s}},n}} ) + Z_{\mathrm{s}}/Z_{\mathrm{v}},$ and $B_{p,n} = - Z_{\mathrm{s}}/Z_{\mathrm{v}}$. In these expressions, *Z*_s_ is the spacer impedance, *h* is the thickness of the spacer, $k_{{\mathrm{v}},n} = \omega _n/c$ and $k_{z{\mathrm{v}},n} = \sqrt {k_{{\mathrm{v}},n}^2 - {\boldsymbol{k}}_n^2}$ are the magnitudes of the wave-vector and its *z*-component in vacuum for the *n*-th harmonic, and similarly $k_{{\mathrm{s}},n} = k_{{\mathrm{v}},n}/Z_{\mathrm{s}}$ and $k_{z{\mathrm{s}},n} = \sqrt {k_{{\mathrm{s}},n}^2 - {\boldsymbol{k}}_n^2}$ are the corresponding quantities in the spacer. The reverse scattered field is given by a double summation over incoming and outgoing frequency harmonics, and a double integral over the respective incoming and outgoing plane-wave components: ${\boldsymbol{E}}_\xi ^{{\mathrm{scatt}},{\mathrm{REV}}}\left( {{\boldsymbol{r}},z,t} \right) = E^{{\mathrm{in}}}{\sum}_{n^{\prime} ,n = - \infty }^\infty {{\int} {d^2} } {\boldsymbol{k}}\prime {\int} {d^2} {\boldsymbol{k}}\left( {2\pi } \right)^{ - 4}\left[ {{\hat{\boldsymbol{e}}}_\xi ^ + \left( {{\boldsymbol{k}}_{n\prime }^\prime } \right) \odot {\hat{\boldsymbol{e}}}_\xi ^ - \left( { - {\boldsymbol{k}}_n} \right)} \right] \times$ $\tilde {\cal{E}}_{\xi ,n\prime }[ - {\boldsymbol{k}}_n,{\boldsymbol{k}}_{n\prime }^\prime ,\omega _{n + n\prime };{\boldsymbol{r}}]\tilde {\cal{E}}_{\xi ,n}[{\boldsymbol{k}}_{{\mathrm{in}}},{\boldsymbol{k}}_n,\omega _n;{\boldsymbol{r}}]e^{i[{\boldsymbol{r}} \cdot {\boldsymbol{k}}_{n\prime }^\prime + zk_{z{\mathrm{v}},n\prime }^\prime - \omega _{n + n\prime }t]}$ (the ⊙ product gives a vector whose components are the product of the Cartesian components of the two polarization unit vectors). For our nonreciprocal beam steering and focusing experiments of Fig. 3b, g and Fig. 4c, d, only the *n* = +1 and *n*′ = −1 terms should be considered, rendering the output frequency equal to *ω*_in_. For Fig. 3c, *n* = *n*′ = +1, and for Fig. 3d, *n* = +1 and *n*′ = 0. The calculated scanning angles $\alpha _{{\mathrm{R}},{\mathrm{L}}}^{{\mathrm{th}}} = {\mathrm{arcos}}\left\{ {\frac{{ - \left( {L_x/2d} \right){\mathrm{tan}}\left( {g_ \pm ^{{\mathrm{th}}}} \right) + \sqrt {{\mathrm{tan}}^2\left( {g_ \pm ^{{\mathrm{th}}}} \right) + \left[ {1 - \left( {L_x/2d} \right)^2} \right]{\mathrm{tan}}^4\left( {g_ \pm ^{{\mathrm{th}}}} \right)} }}{{1 + {\mathrm{tan}}^2\left( {g_ \pm ^{{\mathrm{th}}}} \right)}}} \right\}$ in Fig. 4c are the angles formed between STMM vertical and the crossings of the main directions of emission from the right and left edges of the STMM with the measurement arc. Here, the “+” (“−”) sign corresponds to $\alpha _{\mathrm{R}}^{{\mathrm{th}}}$ $\left( {\alpha _{\mathrm{L}}^{{\mathrm{th}}}} \right)$, $g_ \pm ^{{\mathrm{th}}} = {\mathrm{arccos}}[1 \pm (\omega _{{\mathrm{in}}}/c)^{ - 1}(d/dx)\varphi _{\bar n = + 1}^{{\mathrm{focus}}}(\!\pm L_x/2)],$ *L*_*x*_ is the size of the STMM along the *x*-direction, and *d* is the distance between the detector and the center of the STMM.

## Supplementary information

Supplementary Information

## Data Availability

The data that support the plots within this paper and other findings of this study are available from the corresponding authors upon reasonable request.

## Code availability

The codes are available from the corresponding authors upon reasonable request.
