# Supplementary material for: Surface-wave-assisted nonreciprocity in spatio-temporally modulated metasurfaces
Source: Nat Commun. 2020 Mar 19;11:1469. doi: 10.1038/s41467-020-15273-1 (PMC7081213; doi:10.1038/s41467-020-15273-1)
Supplement: Supplementary file 1 — Supplementary Information [file 41467_2020_15273_MOESM1_ESM.pdf]

Supplementary Information: Surface-Wave-Assisted Nonreciprocity in Spatio-Temporally Modulated Metasurfaces

Andrew E. Cardin[1, 2], Sinhara R. Silva[1], Shai. R. Vardeny[1], Willie J. Padilla[2], Avadh Saxena[3], Antoinette J. Taylor[1], Wilton J. M. Kort-Kamp[3], Hou-Tong Chen[1], Diego A. R. Dalvit[3]*, and Abul K. Azad[1]*

[1] *Center for Integrated Nanotechnologies, Los Alamos National Laboratory, Los Alamos, New Mexico 87545, USA*

[2] *Department of Electrical and Computer Engineering, Duke University, Durham, North Carolina 27708, USA*

[3] *Theoretical Division, Los Alamos National Laboratory, Los Alamos, New Mexico 87545, USA*

* Corresponding authors: A.A.: aazad@lanl.gov and D.D.: dalvit@lanl.gov

Supplementary Note 1: Generalized Bloch-Floquet Theory for Arbitrary Phase Distributions. We describe the optical response of the unmodulated metasurface reflectarray using an effective 2D complex conductivity $\sigma_{\text{unmod}}(\omega)$ for the sub-wavelength resonators loaded with varactors, uniformly biased with the same external voltage (Supplementary Fig. 1). This conductivity (Supplementary Fig. 3a,b) is extracted from reflectivity measurements (Supplementary Fig. 2a,b) and standard Fresnel equations for multilayered systems: in our case, the 2D conductivity sheet, the spacer, and a perfectly electric conducting ground plane (Fig. 1d in the main text). The modulation of the capacitance of each resonator results in a modulation of the conductivity, $\sigma(\boldsymbol{r},\omega;t)=\sigma_{\text{op}}(\omega)+[\Delta\sigma_R(\omega)+i\Delta\sigma_I(\omega)]\sin[\varphi(\boldsymbol{r})-\Omega\, t]$. Here $\sigma_{\text{op}}(\omega)$ is $\sigma_{\text{unmod}}(\omega)$ at $V=2$ V. From the data reported in Supplementary Fig. 2a,b we estimate the real and imaginary parts of the conductivity modulations as $\Delta\sigma_{R,I}(\omega)=\left|\sigma_{R,I}^{V=3\text{V}}(\omega)-\sigma_{R,I}^{V=1\text{V}}(\omega)\right|/2$ (Supplementary Fig. 3c). At $\omega=\omega_{\text{in}}=2\pi\times 6.9\ \text{GHz}$, we obtain $\Delta\sigma_R(\omega_{\text{in}})/|\sigma_{\text{op}}(\omega_{\text{in}})|=1.6\times10^{-2}$ and $\Delta\sigma_I(\omega_{\text{in}})/|\sigma_{\text{op}}(\omega_{\text{in}})|=3.2\times10^{-2}$. Finally, in Supplementary Fig. 3d we check the linearity between voltage modulation and the resulting conductivity modulation. In the linear regime, we expect that $\sigma_{\text{op}}(\omega)$ should be approximately equal to the average $[\sigma^{V=3\text{V}}(\omega)+\sigma^{V=1\text{V}}(\omega)]/2$. Around $\omega=2\pi\times 6.6$ GHz, linearity holds.

When a linearly polarized plane wave $\boldsymbol{E}_\xi^{\text{in}}(\boldsymbol{r},z,t)=\hat{\boldsymbol{e}}_\xi^-(\boldsymbol{k}_{\text{in}})\,E^{\text{in}}\,e^{i(\boldsymbol{r}\cdot\boldsymbol{k}_{\text{in}}-z\,k_{z\text{v,in}}-\omega_{\text{in}}t)}$ impinges on the STMM, the reflected field is given as

$$\boldsymbol{E}_\xi^{\text{refl}}(\boldsymbol{r},z,t)=E^{\text{in}}\sum_{n=-\infty}^{\infty}\int\frac{d^2\boldsymbol{k}}{(2\pi)^2}\,\hat{\boldsymbol{e}}_\xi^+(\boldsymbol{k}_n)\,\widetilde{\mathcal{E}}_{\xi,n}[\boldsymbol{k}_{\text{in}},\boldsymbol{k}_n,\omega_n;\boldsymbol{r}]\,e^{i\,(\boldsymbol{r}\cdot\,\boldsymbol{k}_n+z\,k_{z\text{v},n}-\omega_n t)},\qquad \text{(S1)}$$

where E^{in} is the amplitude of the incoming field, $\boldsymbol{k}_{\text{in}}$ and $k_{z\text{v},0}$ are respectively the projections of the incoming wave-vector on the *x*-*y* plane and the *z*-axis. Furthermore, ξ indicates the field's *s* or *p* polarization, $\hat{\boldsymbol{e}}_s^\pm(\boldsymbol{k})=(\hat{\boldsymbol{z}}\times\boldsymbol{k})/|\boldsymbol{k}|$ and $\hat{\boldsymbol{e}}_p^\pm(\boldsymbol{k})=(\hat{e}_s^\pm(\boldsymbol{k})\times\boldsymbol{K}^\pm)/|\boldsymbol{K}^\pm|$ are the *s* and *p*

polarization unit vectors, $\boldsymbol{K}^{\pm} = \boldsymbol{k} \pm k_z\, \hat{\boldsymbol{z}}$, and the + and – signs correspond to waves propagating in the positive and negative z directions, respectively. Also, $\omega_n = \omega_{\rm in} + n\,\Omega$, and $\boldsymbol{k}_n = \boldsymbol{k} + n\,\nabla\varphi(\boldsymbol{r})$. Similarly, the reflected magnetic field takes the form

$$\boldsymbol{H}_{\xi}^{\rm refl}(\boldsymbol{r}, z, t) = \pm \frac{E^{\rm in}}{Z_{\rm v}} \sum_{n=-\infty}^{\infty} \int \frac{d^2\boldsymbol{k}}{(2\pi)^2}\, \hat{\boldsymbol{e}}^{+}_{\xi' \neq \xi}(\boldsymbol{k}_n)\, \widetilde{\mathcal{E}}_{\xi,n}[\boldsymbol{k}_{\rm in}, \boldsymbol{k}_n, \omega_n; \boldsymbol{r}]\, e^{i\,(\boldsymbol{r}\cdot\,\boldsymbol{k}_n + z\,k_{z{\rm v},n} - \omega_n t)}, \quad \text{(S2)}$$

where $Z_{\rm v}$ is the vacuum impedance, and the '+' and '–' in front of the expression in the right-hand-side respectively correspond to $\xi = p$ and $\xi = s$ polarizations. We employ a local derivative expansion of the 2D phase distribution, $\varphi(\boldsymbol{r}) \approx \varphi(\boldsymbol{r}_0) + (\boldsymbol{r} - \boldsymbol{r}_0)\cdot\nabla\varphi(\boldsymbol{r}_0)$, where $\boldsymbol{r}_0$ is close to $\boldsymbol{r}$ and we take the limit $\boldsymbol{r}_0 \to \boldsymbol{r}$ at the end of the calculations. Our approach is valid for smooth but otherwise arbitrary spatial phase distributions. The field amplitude $\widetilde{\mathcal{E}}_{\xi,n}[\boldsymbol{k}_{\rm in}, \boldsymbol{k}_n, \omega_n; \boldsymbol{r}]$ depends locally on both $\varphi(\boldsymbol{r})$ and $\nabla\varphi(\boldsymbol{r})$, and is given by

$$\widetilde{\mathcal{E}}_{\xi,n}[\boldsymbol{k}_{\rm in}, \boldsymbol{k}_n, \omega_n; \boldsymbol{r}] = R_{\xi,n}(\boldsymbol{k}, \omega_{\rm in}; \boldsymbol{r})$$

$$\times \left\{ \delta_{n,0}\, \delta(\boldsymbol{k} - \boldsymbol{k}_{\rm in}) + \left(1 - \delta_{n,0}\right) \frac{{\rm FT}[e^{i\,{\rm sg}(n)\varphi(\boldsymbol{r})}]}{e^{i{\rm sg}(n)[\varphi(\boldsymbol{r}) - \boldsymbol{r}\cdot\nabla\varphi(\boldsymbol{r})]}} \left[\frac{R_{\xi,n-{\rm sg}(n)}(\boldsymbol{k}_{\rm in}, \omega_{\rm in}; \boldsymbol{r}) \pm \delta_{|n|,1}}{R_{\xi,n-{\rm sg}(n)}(\boldsymbol{k}, \omega_{\rm in}; \boldsymbol{r}) \pm \delta_{|n|,1}} \right] \right\}, \quad \text{(S3)}$$

where the '+' and '–' correspond to $\xi = s$ and $\xi = p$ polarizations, respectively. In Eq. (S3), $R_{\xi,n}(\boldsymbol{k}, \omega_{\rm in}; \boldsymbol{r})$ is the position-dependent reflection coefficient that describes the scattering process $(\boldsymbol{k}, \omega_{\rm in}) \to (\boldsymbol{k}_n, \omega_n)$ at $\boldsymbol{r}$, and the 2D Fourier transform ${\rm FT}[e^{i\,{\rm sg}(n)\varphi(\boldsymbol{r})}]$ should be evaluated at $\boldsymbol{k} - \boldsymbol{k}_{\rm in} + {\rm sg}(n)\,\nabla\varphi(\boldsymbol{r})$.

For the steering case $\varphi^{\rm steer}(\boldsymbol{r}) = \boldsymbol{\beta}\cdot\boldsymbol{r}$, the Fourier transform is ${\rm FT}[e^{i\,{\rm sg}(n)\varphi^{\rm steer}(\boldsymbol{r})}]_{\boldsymbol{k}-\boldsymbol{k}_{\rm in}+{\rm sg}(n)\boldsymbol{\beta}} = (2\pi)^2\delta(\boldsymbol{k} - \boldsymbol{k}_{\rm in})$, and the derivative expansion is exact. For the 1D focusing case at the $\bar{n}$ frequency harmonic, and for normal incidence along the x-direction, $\boldsymbol{k}_{\rm in} = k_{{\rm in},x}\hat{\boldsymbol{x}}$, the corresponding Fourier transform is

$$\mathrm{FT}[e^{i\,\mathrm{sg}(n)\varphi_{\bar{n}}^{\mathrm{focus}}(x)}]_{\boldsymbol{k}-\boldsymbol{k}_{\mathrm{in}}+\mathrm{sg}(n)\frac{d}{dx}\varphi_{\bar{n}}^{\mathrm{focus}}(\boldsymbol{k}_{\mathrm{in}},\omega_{\mathrm{in}};x)}$$

$$= -2\,\pi^2\delta(k_y)\, z_f\, |a|\, e^{i\,\mathrm{sg}(n)\, z_f\,|a|}\, e^{-i\,x_f\left[|b|-\frac{\mathrm{sg}(n)}{\bar{n}}k_{\mathrm{in},x}\right]}\, F(a,b), \tag{S4}$$

where $a = \omega_{\mathrm{in}}/c + n\,\Omega/c\,\bar{n},\;\; b = k_x - k_{\mathrm{in},x} + \mathrm{sg}(n)\,[\,k_{\mathrm{in},x}/\bar{n} + (d/dx)\,\varphi_{\bar{n}}^{\mathrm{focus}}(\boldsymbol{k}_{\mathrm{in}},\omega_{\mathrm{in}};x)]$,

and

$$F(a,b) = \begin{cases} \dfrac{H_1^{(2)}\left[z_f\sqrt{a^2-b^2}\right]}{\sqrt{a^2-b^2}}, & |b|<|a| \\ -\dfrac{2i}{\pi}\,\dfrac{K_1\left[z_f\sqrt{b^2-a^2}\right]}{\sqrt{b^2-a^2}}, & |a|<|b|. \end{cases} \tag{S5}$$

Here, $H_\nu^{(2)}$ is the Hankel function of second kind and K_ν is the modified Bessel function of the second kind.

Supplementary Note 2: Position-dependent Reflection Coefficients. The reflection coefficients $R_{\xi,n}(\boldsymbol{k},\omega_{\mathrm{in}};\boldsymbol{r})$ can be obtained by using the local derivative expansion in Maxwell equations and solving numerically (or via continued fractions) an infinite set of coupled equations, $\eta^{+}_{\xi,n-1}\,\mathcal{R}_{\xi,n-1} + A_{\xi,n}\,\mathcal{R}_{\xi,n} + \eta^{-}_{\xi,n+1}\,\mathcal{R}_{\xi,n+1} = 2\,B_{\xi,n}\,\delta_{n,0}$. For *s*-polarization,

$$\mathcal{R}_{s,n}(\boldsymbol{k},\omega_{\mathrm{in}};\boldsymbol{r}) = R_{s,n}(\boldsymbol{k},\omega_{\mathrm{in}};\boldsymbol{r}) + \delta_{n,0},$$

$$\eta^{\pm}_{s,n} = \mp\, i\,\frac{Z_{\mathrm{s}}}{2}\Delta\sigma(\omega_n)e^{\pm i\,[\varphi(\boldsymbol{r})-\boldsymbol{r}\cdot\nabla\varphi(\boldsymbol{r})]},$$

$$A_{s,n} = Z_{\mathrm{s}}\,\sigma_{\mathrm{op}}(\omega_n) + i\cot(h\,k_{z\mathrm{s},n})\,\frac{k_{z\mathrm{s},n}}{k_{\mathrm{s},n}} + \frac{Z_{\mathrm{s}}}{Z_{\mathrm{v}}}\,\frac{k_{z\mathrm{v},n}}{k_{\mathrm{v},n}},$$

$$B_{s,n} = \frac{Z_{\mathrm{s}}}{Z_{\mathrm{v}}}\frac{k_{z\mathrm{v},n}}{k_{\mathrm{v},n}}. \tag{S6}$$

For *p*-polarization,

$$\mathcal{R}_{p,n}(\boldsymbol{k},\omega_{\mathrm{in}};\boldsymbol{r}) = R_{p,n}(\boldsymbol{k},\omega_{\mathrm{in}};\boldsymbol{r}) - \delta_{n,0},$$

$$\eta_{p,n}^{\pm} = \mp i \, \frac{Z_{\rm s}}{2} \frac{k_{z{\rm v},n}}{k_{{\rm v},n}} \Delta\sigma(\omega_n) e^{\pm i \, [\varphi(\boldsymbol{r}) - \boldsymbol{r}\cdot\nabla\varphi(\boldsymbol{r})]},$$

$$A_{p,n} = Z_{\rm s} \, \sigma_{\rm op}(\omega_n) \, \frac{k_{z{\rm v},n}}{k_{{\rm v},n}} + i \cot\!\left(h \, k_{z{\rm s},n}\right) \frac{k_{z{\rm v},n} \, k_{{\rm s},n}}{k_{{\rm v},n} \, k_{z{\rm s},n}} + \frac{Z_{\rm s}}{Z_{\rm v}},$$

$$B_{p,n} = -\frac{Z_{\rm s}}{Z_{\rm v}}. \tag{S7}$$

In these expressions, $Z_{\rm s}$ is the spacer impedance, h is the thickness of the spacer, $k_{{\rm v},n} = \omega_n/c$ and $k_{z{\rm v},n} = \sqrt{k_{{\rm v},n}^2 - \boldsymbol{k}_n^2}$ are the magnitudes of the wave-vector and its z-component in vacuum for the n-th harmonic, and similarly $k_{{\rm s},n} = k_{{\rm v},n}/Z_{\rm s}$ and $k_{z{\rm s},n} = \sqrt{k_{{\rm s},n}^2 - \boldsymbol{k}_n^2}$ are the corresponding quantities in the spacer. All plots shown in Supplementary Figs. 4-6 use the exact solutions to Eq. (S7).

Supplementary Fig. 4 reports the amplitude of the steering p-reflection coefficients $R_{p,n=0,\pm1}^{\rm steer}(k_{{\rm in},x}, \omega_{\rm in}; x)$ as a function of the incoming frequency for a β_x =44 m^{-1} phase gradient. The amplitude for $n = 0$ is much larger than those for $n = \pm 1$ (that have identical amplitude at normal incidence) in the whole range of frequencies, except close to the resonance $\omega_{\rm res} = 2\pi \times 6.6$ GHz where the unmodulated reflectivity has a minimum for $V_{\rm op} = 2$ V (Supplementary Fig. 2). The amplitudes of the reflection coefficients at resonance are $\left|R_{p,n=0}^{\rm steer}(0, \omega_{\rm res})\right| = 0.016$ and $\left|R_{p,n=\pm1}^{\rm steer}(0, \omega_{\rm res})\right| = 0.011$ for normal incidence ($\boldsymbol{k}_{\rm in} = 0$). At the input frequency $\omega_{\rm in}$ we obtain $\left|R_{p,n=0}^{\rm steer}(0, \omega_{\rm in})\right| = 0.186$ and $\left|R_{p,n=\pm1}^{\rm steer}(0, \omega_{\rm in})\right| = 0.026$. One might have expected that close to the resonance the conversion efficiency to the $n = \pm 1$ harmonics would be larger than the one for $n = 0$, which is clearly not the case. In order to explain the reason for this, it is convenient to analyze the solutions for the p-polarized reflection coefficients. Since in our experiment we are well within the perturbative regime $\Delta\sigma_{R,I}(\omega_{\rm in}) \ll |\sigma_{\rm op}(\omega_{\rm in})|$ (see Supplementary Fig. 3c), we can use perturbation theory to solve Eq. (S7). For the $n = 0, \pm 1$ harmonics we get

$$R_{p,0}^{\mathrm{pert}}(\boldsymbol{k},\omega_{\mathrm{in}};\boldsymbol{r}) \approx 1 - \frac{2\frac{Z_{\mathrm{s}}}{Z_{\mathrm{v}}}}{A_{p,0} - \left(\frac{\eta_{p,0}^{+}\,\eta_{p,+1}^{-}}{A_{p,+1}} + \frac{\eta_{p,0}^{-}\,\eta_{p,-1}^{+}}{A_{p,-1}}\right)}, \tag{S8}$$

and

$$R_{p,\pm 1}^{\mathrm{pert}}(\boldsymbol{k},\omega_{\mathrm{in}};\boldsymbol{r}) \approx -\frac{\eta_{p,0}^{\pm}}{A_{p,\pm 1} - \frac{\eta_{p,\pm 1}^{\pm}\,\eta_{p,\pm 2}^{\mp}}{A_{p,\pm 2}}}\left[R_{p,n=0}^{\mathrm{pert}}(\boldsymbol{k},\omega_{\mathrm{in}};\boldsymbol{r}) - 1\right]. \tag{S9}$$

In the unmodulated case, our structure is impedance matched ($A_{p,0} \approx 2\,Z_{\mathrm{s}}/Z_{\mathrm{v}}$) at ω_{res}, resulting in almost perfect absorption. When modulation is on, the term in parenthesis in the denominator of Eq. (S8) is in general a small correction to A_0 because it is quadractic in η's. Therefore, in Eq. (S9) we can approximate $R_{p,n=0}^{\mathrm{pert}}(\boldsymbol{k},\omega_{\mathrm{in}};\boldsymbol{r}) - 1 \approx -1$, and note that its prefactor is $\sim |\eta_{p,\pm 1}^{\pm}|/(Z_{\mathrm{s}}/Z_{\mathrm{v}}) \ll 1$ for weak modulation. This results in small conversion efficiency for the $n = \pm 1$ harmonics.

In Supplementary Fig. 5a we show the amplitude of the focusing *p*-reflection coefficients $R_{p,\bar{n},n}^{\mathrm{focus}}(k_{\mathrm{in},x},\omega_{\mathrm{in}};x)$ as a function of position along the STMM for the case $\bar{n} = +1$, $n = +1, +2, +3$ harmonics, and off-center focusing $x_f = 6$ cm, $z_f = 15$ cm. When $|x - x_f| \ll z_f$, the amplitude of the reflection coefficient for $n = +1$ has a parabolic-like shape, and it asymptotes to a constant value for $|x - x_f| \gg z_f$. The other two harmonics show a much smaller conversion efficiency. In Supplementary Fig. 5b we show the respective phases and observe that only the $n = +1$ harmonic has a phase distribution that resembles the parabolic focusing phase, while the phase distributions for $n = +2, +3$ substantially depart from it. This is expected since the focusing distribution is designed for $\bar{n} = +1$. However, note that all three phase distributions are concave, while the phase distribution $\mathrm{sg}(\bar{n})\varphi_{\bar{n}}^{\mathrm{focus}}(x)$ for focusing is always convex for any value of $\bar{n}$ (see

Supplementary Fig. 5c,d). In the next Supplementary Note 3 we explain the reason behind this difference.

Supplementary Note 3: Total Reflection Coefficient. The total reflection coefficient $R_{\xi,n}^{\text{tot}}(\boldsymbol{k}_{\text{in}}, \omega_{\text{in}}; \boldsymbol{r})$ is given by the ratio between the amplitude of the n-th frequency harmonic of the reflected field (*i.e.*, the modulus of the integral in Eq. (S1) after dropping the polarization unit vectors in the integrand and setting $z = 0$), and the incident field amplitude also at $z = 0$. For the case of beam steering, the total reflection coefficient for the n-th harmonic is $R_{\xi,n}^{\text{tot,steer}}(\boldsymbol{k}_{\text{in}}, \omega_n; \boldsymbol{r}) = |R_{\xi,n}^{\text{steer}}(\boldsymbol{k}_{\text{in}}, \omega_{\text{in}})|\ e^{i\,n\,\boldsymbol{\beta}\cdot\boldsymbol{r}}$, where its amplitude is position-independent and its phase is precisely $n\ \varphi^{\text{steer}}(\boldsymbol{r})$. For focusing, the total reflection coefficient depends both on the frequency harmonic $\bar{n}$ for which the focusing phase distribution is designed, and on the frequency harmonic n that one intends to focus. $R_{\xi,\bar{n},n}^{\text{tot,focus}}(k_{\text{in},x}, \omega_{\text{in}}; x) = |R_{\xi,\bar{n},n}^{\text{tot,focus}}(k_{\text{in},x}, \omega_{\text{in}}; x)|\ e^{i\,\text{sg}(\bar{n})\varphi_{\bar{n}}^{\text{focus}}(x)}$ has a position-dependent amplitude and a phase given by Eq. (3) of the main text (after an irrelevant global phase shift is subtracted). In Supplementary Fig. 6a we show the amplitude and phase of $R_{\xi,\bar{n},n}^{\text{tot,focus}}(k_{\text{in},x}, \omega_{\text{in}}; x)$ as a function of position along the metasurface for $n = \bar{n} = +1$. The phase of the total reflection coefficient is equal to $\text{sg}(\bar{n})\varphi_{\bar{n}}^{\text{focus}}(x)$. In this case, the phases match after an irrelevant global phase shift. Next we compare the phase distributions of the reflection coefficient $R_{\xi,\bar{n},n}^{\text{focus}}(k_{\text{in},x}, \omega_{\text{in}}; x)$ with that of the total reflection coefficient $R_{\xi,\bar{n},n}^{\text{tot,focus}}(k_{\text{in},x}, \omega_{\text{in}}; x)$ for the case $n = \bar{n} = +1$, and on- (Supplementary Fig. 6b) and off-axis (Supplementary Fig. 6c) focusing. In both cases, the two distributions do not match (not even after a global phase shift), in fact they have opposite concavity. The reason is that the phase of the full reflected field in Eq. (S1) results only after

performing the momentum integration. The field amplitude $\tilde{\mathcal{E}}_{\xi,n}[\boldsymbol{k}_{\text{in}}, \boldsymbol{k}_n, \omega_n; \boldsymbol{r}]$ is not just proportional to $R^{\text{focus}}_{\xi,\bar{n},n}(\boldsymbol{k}_{\text{in}}, \omega_{\text{in}}; x)$, but contains other factors, in particular the Fourier transform $\text{FT}[\, e^{i\,\text{sg}(\bar{n})\varphi^{\text{focus}}_{\bar{n}}(x)}\,]$ (Eq. (S3)). Hence, the phase of the total reflection coefficient is not simply the phase of the reflection coefficient $R^{\text{focus}}_{\xi,\bar{n},n}(\boldsymbol{k}_{\text{in}}, \omega_{\text{in}}; x)$. It is possible to analytically show that, using Eq. (S3) and performing the $\boldsymbol{k}$ integral in Eq. (S1), the phase of $R^{\text{tot,focus}}_{\xi,\bar{n},n}(k_{\text{in},x}, \omega_{\text{in}}; x)$ is indeed $\text{sg}(\bar{n})\varphi^{\text{focus}}_{\bar{n}}(x)$, as shown in Supplementary Fig. 6a. Furthermore, the phase distribution of the total reflection coefficient turns out to be convex for all values of $\bar{n}$ and n, while that of the reflection coefficients are always concave for all values of $\bar{n}$ and n.

Finally, we emphasize that it is not possible to focus the fundamental frequency harmonic $n = 0$ when spatio-temporal modulation is applied. As mentioned in the main text, Eq. (3) does not hold for $\bar{n} = 0$. The fundamental frequency harmonic undergoes frequency-conserving specular reflection despite the presence of the spatio-temporal focusing modulation. However, it is still possible to focus the $n = 0$ frequency harmonic with STMMs by switching off the temporal modulation, *i.e.*, setting Ω to zero, and keeping only the spatial modulation on. In this case, the required voltage modulation is $V(\boldsymbol{r}) = V_{\text{op}} + \Delta_V \sin[\varphi^{\text{focus}}_{\text{static}}(\boldsymbol{r})]$, where $\varphi^{\text{focus}}_{\text{static}}(\boldsymbol{r}) = -\left[\frac{\omega_{\text{in}}}{c}\left(|\boldsymbol{r} - \boldsymbol{R}_f| - z_f\right) + \boldsymbol{k}_{\text{in}} \cdot \left(\boldsymbol{r} - \boldsymbol{R}_f\right)\right]$ Importantly, this phase distribution is the standard one used for static focusing, and is different from that required for dynamical focusing (Eq. 3). Time-inversion symmetry is clearly preserved under this static modulation, and therefore Lorentz reciprocity is not broken.

Supplementary Note 4: Finite-sized STMMs. We should emphasize that the above theory considers an infinite-sized STMM. When the modulation takes place only over a finite-sized

region of the metasurface, it is straightforward to generalize our theory by simply making the replacement $\Delta\sigma_{R,I}(\omega) \rightarrow \tilde{\theta}(\boldsymbol{r})\, \Delta\sigma_{R,I}(\omega)$, where $\tilde{\theta}(\boldsymbol{r}) = 1$ if $\boldsymbol{r}$ belongs to the modulated region and is zero otherwise. Making this modification in our generalized Bloch-Floquet analytical approach, all results for the reflected field remain the same after changing the Fourier transform in Eq. (S3) as $\mathrm{FT}\left[e^{i\,\mathrm{sg}(n)\varphi(r)}\right] \rightarrow \mathrm{FT}\left[\tilde{\theta}(\boldsymbol{r})\, e^{i\,\mathrm{sg}(n)\varphi(r)}\right]$. It is important to note that in this approach we assume that the metasurface contains a spatio-temporally modulated region ($\tilde{\theta}(\boldsymbol{r}) = 1$), and another one where no modulation takes place ($\tilde{\theta}(\boldsymbol{r}) = 0$). The unmodulated region still interacts with incoming waves, but it does not cause changes in the momentum or frequency harmonics contents of the incoming field, and simply reflects it specularly conserving its frequency. The full reflected field is then the superposition of the part reflected from the unmodulated region and that reflected from the finite-sized STMM. Given that the former does not undergo momentum nor frequency conversion, but the latter does, it is straightforward to distinguish between the two.

As examples of this extension of our generalized Bloch-Floquet theory to finite-sized STMMs, we first consider the steering functionality $\varphi^{\mathrm{steer}}(x) = \beta x$. We obtain for the Fourier transform $\mathrm{FT}\left[\tilde{\theta}(\boldsymbol{r})\, e^{\pm i\,\varphi^{\mathrm{steer}}(x)}\right]_{\boldsymbol{\kappa}} = (2\pi)\delta(\kappa_y) L_x \mathrm{sinc}[(L_x/2)\,(\pm\beta - \kappa_x)]$, where $\mathrm{sinc}(x) = \sin(x)/x$ is the sinc function and L_x along the relevant x-direction. For the case of focusing $\varphi^{\mathrm{focus}}(x) \equiv \varphi^{\mathrm{focus}}_{\bar{n}}(x)$, $\mathrm{FT}\left[\tilde{\theta}(\boldsymbol{r})\, e^{\pm i\,\varphi^{\mathrm{focus}}(x)}\right]_{\boldsymbol{\kappa}}$ does not have a closed analytical form. In contrast to steering, this Fourier transform is a complex quantity, and both its real and imaginary parts have several peaks and dips with non-negligible amplitude. For on-axis focusing it is an even function of the argument $\boldsymbol{\kappa}$, but for off-axis focusing it does not have a well-defined parity. We denote the values of κ_x for which $\mathrm{ReFT}\left[\tilde{\theta}(\boldsymbol{r})\, e^{\pm i\,\varphi^{\mathrm{focus}}(x)}\right]_{\boldsymbol{\kappa}}$ has zero derivate as $\kappa^{\pm}_{R,j}$, and $\kappa^{\pm}_{I,j}$ for $\mathrm{ImFT}\left[\tilde{\theta}(\boldsymbol{r})\, e^{\pm i\,\varphi^{\mathrm{focus}}(x)}\right]_{\boldsymbol{\kappa}}$. Given that $\mathrm{ReFT}\left[\tilde{\theta}(\boldsymbol{r})\, e^{+i\,\varphi^{\mathrm{focus}}(x)}\right]_{\boldsymbol{\kappa}} = \mathrm{ReFT}\left[\tilde{\theta}(\boldsymbol{r})\, e^{-i\,\varphi^{\mathrm{focus}}(x)}\right]_{-\boldsymbol{\kappa}}$

and $\mathrm{ImFT}\left[\tilde{\theta}(\boldsymbol{r})\, e^{+i\,\varphi^{\mathrm{focus}}(x)}\right]_{\boldsymbol{\kappa}} = -\mathrm{ImFT}\left[\tilde{\theta}(\boldsymbol{r})\, e^{-i\,\varphi^{\mathrm{focus}}(x)}\right]_{-\boldsymbol{\kappa}}$, those values are related as $\kappa^{+}_{R/I,j} = -\kappa^{-}_{R/I,j}$.

Finally, we evaluate the gain for the dynamical focusing experiments. For the off-axis case, we estimate a gain at $\ell = 13$ cm of 5.24 dB (Fig. 2e). For a finite-sized STMM with parameters of a traditional parabolic dish antenna, $L_x = 45.72$ cm (18 in), focal length of 50 cm, and focal axis at 35°, we estimate a gain at the focal point of 5.65 dB with our flat STMM.

Supplementary Note 5: Nonreciprocity for Arbitrary Wave-Fronts. For the forward process, consider an incoming plane wave $(\boldsymbol{k}_{\mathrm{in}}, \omega_{\mathrm{in}})$ that reflects off the STMM via a phase profile $\varphi(\boldsymbol{r})$ according to Eq. (S1). The corresponding frequency-momentum harmonics amplitude $\widetilde{\mathcal{E}}_{\xi,n}[\boldsymbol{k}_{\mathrm{in}}, \boldsymbol{k}_n, \omega_n; \boldsymbol{r}]$ is proportional to the reflection coefficient $R_{\xi,n}(\boldsymbol{k}, \omega_{\mathrm{in}}; \boldsymbol{r})$, *i.e.,* a scattering process $(\boldsymbol{k}, \omega_{\mathrm{in}}) \rightarrow (\boldsymbol{k}_n, \omega_n)$ at $\boldsymbol{r}$. For the reverse process, the reflected field is sent back onto the STMM, and each of its plane wave components is subjected the same phase distribution $\varphi(\boldsymbol{r})$ that the forward field experienced. We obtain the incoming electric field for the reverse process using time-reversal by replacing $\boldsymbol{k}_n \rightarrow -\boldsymbol{k}_n$ and $k_{\mathrm{zv},n} \rightarrow -k_{\mathrm{zv},n}$ in Eq. (S1), except in the amplitudes $\widetilde{\mathcal{E}}_{\xi,n}$:

$$\boldsymbol{E}_{\xi}^{\mathrm{in,REV}}(\boldsymbol{r}, z, t) = E^{\mathrm{in}} \sum_{n=-\infty}^{\infty} \int \frac{d^2\boldsymbol{k}}{(2\pi)^2}\, \hat{\boldsymbol{e}}_{\xi}^{-}(-\boldsymbol{k}_n)\, \widetilde{\mathcal{E}}_{\xi,n}[\boldsymbol{k}_{\mathrm{in}}, \boldsymbol{k}_n, \omega_n; \boldsymbol{r}]\, e^{i\,(-\boldsymbol{r}\cdot\boldsymbol{k}_n - z\,k_{\mathrm{zv},n} - \omega_n t)}, \quad \text{(S10)}$$

and similarly, for the magnetic field $\boldsymbol{H}_{\xi}^{\mathrm{in,REV}}(\boldsymbol{r}, z, t)$. The reserve outgoing scattered field is given as

$$\boldsymbol{E}_{\xi}^{\mathrm{scatt,REV}}(\boldsymbol{r}, z, t) = E^{\mathrm{in}} \sum_{n'=-\infty}^{\infty} \sum_{n=-\infty}^{\infty} \int \frac{d^2\boldsymbol{k}'}{(2\pi)^2} \int \frac{d^2\boldsymbol{k}}{(2\pi)^2}\, [\hat{\boldsymbol{e}}_{\xi}^{+}(\boldsymbol{k}'_{n'}) \odot \hat{\boldsymbol{e}}_{\xi}^{-}(-\boldsymbol{k}_n)]$$

$$\times\ \widetilde{\mathcal{E}}_{\xi,n'}[-\boldsymbol{k}_n, \boldsymbol{k}'_{n'}, \omega_{n+n'}; \boldsymbol{r}]\ \ \widetilde{\mathcal{E}}_{\xi,n}[\boldsymbol{k}_{\text{in}}, \boldsymbol{k}_n, \omega_n; \boldsymbol{r}]\ e^{i\,[\boldsymbol{r}\cdot \boldsymbol{k}'_{n'} + z\, k'_{z\text{v},n'} - \omega_{n+n'} t]}, \qquad \text{(S11)}$$

that is, a double summation over reverse incoming and outgoing frequency harmonics, and a double integration over the corresponding momenta. Here, $\hat{\boldsymbol{e}}^{+}_{\xi}(\boldsymbol{k}'_{n'}) \odot \hat{\boldsymbol{e}}^{-}_{\xi}(-\boldsymbol{k}_n)$ is a vector whose components are the product of the Cartesian components of $\hat{\boldsymbol{e}}^{+}_{\xi}(\boldsymbol{k}'_{n'})$ and $\hat{\boldsymbol{e}}^{-}_{\xi}(-\boldsymbol{k}_n)$. For the nonreciprocal experiments done in this work, we are only interested in the $n = +1$ and $n' = -1$ terms, corresponding to a frequency up-conversion in the forward process ($\widetilde{\mathcal{E}}_{\xi,+1}[\boldsymbol{k}_{\text{in}}, \boldsymbol{k}_{+1}, \omega_{+1}; \boldsymbol{r}]$ proportional to $R_{\xi,+1}(\boldsymbol{k}, \omega_{\text{in}}; \boldsymbol{r})$) and a frequency down-conversion in the reverse process ($\widetilde{\mathcal{E}}_{\xi,-1}[-\boldsymbol{k}_{+1}, \boldsymbol{k}'_{-1}, \omega_{\text{in}}; \boldsymbol{r}]$ proportional to $R_{\xi,-1}(\boldsymbol{k}', \omega_{+1}; \boldsymbol{r})$), rendering the reverse output frequency equal to ω_{in}. Hence, the scattered field in the reverse process reads

$$\boldsymbol{E}^{\text{scatt,REV}}_{\xi}(\boldsymbol{r}, z, t) = E^{\text{in}} \int \frac{d^2\boldsymbol{k}'}{(2\pi)^2} \int \frac{d^2\boldsymbol{k}}{(2\pi)^2}\ [\hat{\boldsymbol{e}}^{+}_{\xi}(\boldsymbol{k}'_{-1}) \odot \hat{\boldsymbol{e}}^{-}_{\xi}(-\boldsymbol{k}_{+1})]$$

$$\times\ \widetilde{\mathcal{E}}_{\xi,-1}[-\boldsymbol{k}_{+1}, \boldsymbol{k}'_{-1}, \omega_{\text{in}}; \boldsymbol{r}]\ \ \widetilde{\mathcal{E}}_{\xi,+1}[\boldsymbol{k}_{\text{in}}, \boldsymbol{k}_{+1}, \omega_{+1}; \boldsymbol{r}]\ e^{i\,[\boldsymbol{r}\cdot \boldsymbol{k}'_{-1} + z\, k'_{z\text{v},-1} - \omega_{\text{in}} t]}. \qquad \text{(S12)}$$

To get a better grasp of this reverse process, it is convenient to evaluate the Fourier transforms contained in $\widetilde{\mathcal{E}}_{\xi,-1}[-\mathbf{k}_{+1}, \mathbf{k}'_{-1}, \omega_{\text{in}}; \mathbf{r}]$ and $\widetilde{\mathcal{E}}_{\xi,+1}[\mathbf{k}_{\text{in}}, \mathbf{k}_{+1}, \omega_{+1}; \mathbf{r}]$ using the derivative expansion. They respectively give $\text{FT}[e^{i\,\text{sg}(-n)\varphi(\boldsymbol{r})}]_{\boldsymbol{k}'+\boldsymbol{k}} \approx (2\pi)^2\delta(\boldsymbol{k}' + \boldsymbol{k}_{+1})$ and $\text{FT}[e^{i\,\text{sg}(n)\varphi(\boldsymbol{r})}]_{\boldsymbol{k}-\boldsymbol{k}_{\text{in}}+\nabla\varphi(\boldsymbol{r})} \approx (2\pi)^2\delta(\boldsymbol{k} - \boldsymbol{k}_{\text{in}})$. Inserting these relations in Eq. (S12), the reverse scattered field takes the form

$$\boldsymbol{E}^{\text{scatt,REV}}_{\xi}(\boldsymbol{r}, z, t) \approx E^{\text{in}}\, [\hat{\boldsymbol{e}}^{+}_{\xi}(-\boldsymbol{k}_{\text{in}} - 2\nabla\varphi(\boldsymbol{r})) \odot \hat{\boldsymbol{e}}^{-}_{\xi}(-\boldsymbol{k}_{\text{in}} - \nabla\varphi(\boldsymbol{r}))]\ \ R_{\xi,+1}(\boldsymbol{k}_{\text{in}}, \omega_{\text{in}}; \boldsymbol{r})$$

$$\times R_{\xi,-1}(-\boldsymbol{k}_{\text{in}} - \nabla\varphi(\boldsymbol{r}), \omega_{+1}; \boldsymbol{r})\, e^{i\left[\boldsymbol{r}\cdot(-\boldsymbol{k}_{\text{in}} - 2\nabla\varphi(\boldsymbol{r})) + z\sqrt{\left(\frac{\omega_{\text{in}}}{c}\right)^2 - (\boldsymbol{k}_{\text{in}} + 2\nabla\varphi(\boldsymbol{r}))^2} - \omega_{\text{in}} t\right]}. \qquad \text{(S13)}$$

Hence, $\boldsymbol{E}^{\text{scatt,REV}}_{\xi}$ has in-plane momentum components equal to $-\boldsymbol{k}_{\text{in}} - 2\,\nabla\varphi(\boldsymbol{r})$ and one does not recover the time-reversed input beam $(-\boldsymbol{k}_{\text{in}}, \omega_{\text{in}})$. A more rigorous calculation requires to evaluate Eq. (S12) with the exact Fourier transforms. One can still prove that indeed $\boldsymbol{E}^{\text{scatt,REV}}_{\xi}(\boldsymbol{r}, z, t)$ is

not the time-reversed version of the incoming field $\boldsymbol{E}_{\xi}^{\text{in}}(\boldsymbol{r}, z, t)$ in the forward process. Therefore, we have demonstrated the breakdown of Lorentz reciprocity (in the spatial domain) for arbitrary spatial phase distributions using our theoretical approach to STMMs. Similar calculations can be performed to show nonreciprocity for arbitrary wave-fronts in the frequency-domain by modifying Eq. (S12) to take into account the corresponding scattering processes.

In the main text we show a particular scattering process of the integrand of Eq. (S12) for nonreciprocal focusing, corresponding to $\boldsymbol{k} = \boldsymbol{k}_{\text{in}}$ and $\boldsymbol{k}' = -\boldsymbol{k}_{\text{in}} - \nabla\varphi_{\bar{n}=+1}^{\text{focus}}$. We demonstrate that one excites propagative and evanescent waves (Fig. 4b of the main paper). Similar results are found for any arbitrary choice of $\boldsymbol{k}$ and $\boldsymbol{k}'$, and the total reverse scattered field given the double integration in Eq. (S12) results in extreme breakdown of Lorentz reciprocity.

Supplementary Note 6: Extreme Nonreciprocity in Focusing STMMs. In the case of beam steering $\varphi^{\text{steer}}(x) = \beta x$, we know that the Fourier transforms contained in Eq. (S12) give delta functions, and there is a single in-plane momentum for reverse scattering is $-k_{\text{in},x} - 2\beta$. When $|\beta| > \omega_{\text{in}}/2c$ in forward scattering, only surface modes are excited in reverse scattering, that is, the extreme nonreciprocal regime. For the focusing case $\varphi^{\text{focus}}(x) \equiv \varphi_{\bar{n}=+1}^{\text{focus}}(x)$, several reverse scattering paths are possible. We first analyze the pathway $(-k_{\text{in},x} - \varphi'^{\text{focus}}(x), \omega_{\text{in}} + \Omega) \rightarrow (-k_{\text{in},x} - 2\varphi'^{\text{focus}}(x), \omega_{\text{in}})$ in the integrand of Eq. (S11), analogous to the process occurring in beam steering. In a central region where $|x - x_f| < \delta x$, $k_{zv,-1}^{\text{ref,REV}} = \left[(\omega_{\text{in}}/c)^2 - |k_{\text{in},x} + 2\varphi'^{\text{focus}}(x)|^2\right]^{1/2}$ is real (propagative modes), while in the outer region $|x - x_f| > \delta x$ surface modes are excited ($k_{zv,-1}^{\text{ref,REV}}$ is purely imaginary). These latter modes move away from the central

region along the STMM, and rapidly decay (Fig. 4b). The boundary δx is determined by the equation

$$(\omega_{\text{in}}/c)^2 - (k_{\text{in},x} + 2\varphi'^{\text{focus}}(x_f + \delta x))^2 = 0. \quad \text{(S14)}$$

In the limit $\Omega \ll \omega_{\text{in}}$, which holds in our experiments, the solution of this equation is $\delta x = z_f \left\{ \left[1/2 + 3\,(\,\omega_{\text{in}}/c)^{-1} k_{\text{in},x} \right]^{-2} - 1 \right\}^{-1/2} = z_f/\sqrt{3}$, where in the last equality we assumed normal incidence. The boundary between the two regions shows peaks in the electromagnetic field and Poynting vector spatial profiles. These peaks concentrate at $x_\pm \equiv x_f \pm \delta x$ on the STMM and are radiated away from it, forming a "ribbon" of propagative modes that is orthogonal to the STMM (Fig. 4b). Other scattering processes in the integrand of Eq. (S12) result in similar ribbon-like structures, centered at x_f and of varying widths and heights of the peaks. The integration over all these processes according to Eq. (S12) produces a reversed scattered field from the infinite-sized STMM that shows extreme breakdown of Lorentz reciprocity, with the generation of both low-power propagative waves and evanescent surface modes, in stark contrast to the original input field in the forward process.

Supplementary Note 7: Extreme Nonreciprocity in Beam Steering and Focusing for Finite-sized STMMs. To analyze the structure of the emitted power from the finite-sized STMM in reverse scattering, both into the near- and far-field zones, we compute the time-averaged Poynting vector $\overline{\boldsymbol{S}} = (1/2)\text{Re}[\boldsymbol{E} \times \boldsymbol{H}^*]$, where $\boldsymbol{E}$ is the electric field in Eq. (S12), $\boldsymbol{H}$ is the corresponding magnetic field, and '×' means cross-product between vectors (not to be confused with other instances where the same symbol appears). For p-polarization and $n = \overline{n} = +1$, it is given by

$$\overline{\boldsymbol{S}}_p(x,z) = \int \frac{dk'_x}{2\pi} \int \frac{dk''_x}{2\pi}\, \overline{\boldsymbol{S}}_p(k'_x, k''_x; x, z)$$

$$= \frac{(E^{\rm in})^2}{2\,Z_{\rm v}} \int \frac{dk'_x}{2\pi} \int \frac{dk''_x}{2\pi} \;{\rm Re}\Big\{ e^{i\,[x\,(k'_{x,-1} - k''_{x,-1}) + z\,(k'_{z{\rm v},-1} - k''_{z{\rm v},-1})]}\, \boldsymbol{A}_p(k'_{x,-1}) \times \boldsymbol{B}^*_p(k''_{x,-1}) \Big\}, \quad \text{(S15)}$$

where '*' means complex conjugate, and the integrals over y-components of the momenta in Eq. (S12) have already been performed using the assumed translational invariance along the y-direction. The factors in Eq. (S15) depending on k'_x stem from the reverse scattered $\boldsymbol{E}$ field and the ones depending on k''_x stem from the corresponding $\boldsymbol{H}^*$ field. Also, $\boldsymbol{A}_p(k'_{x,-1}) = \hat{\boldsymbol{e}}^+_p(k'_{x,-1}) \odot \widetilde{\boldsymbol{F}}_p(k'_{x,-1})$ is a vector whose components are the product of the Cartesian components of $\hat{\boldsymbol{e}}^+_p(k'_{x,-1})$ and $\widetilde{\boldsymbol{F}}_p(k'_{x,-1})$, and arises from the electric field. The vector $\widetilde{\boldsymbol{F}}_p(k'_{x,-1})$ is defined as

$$\widetilde{\boldsymbol{F}}_p(k'_{x,-1}) = C_p(k'_{x,-1}; x)$$

$$\times \int \frac{d\bar{k}}{2\pi}\, \hat{\boldsymbol{e}}^-_p(\bar{k}_{+1}) {\rm FT}\big[\tilde{\theta}(u)\, e^{-i\,\varphi(u)}\big]_{k'_{x,-1}+\bar{k}_{+1}} {\rm FT}\big[\tilde{\theta}(u)\, e^{+i\,\varphi(u)}\big]_{\bar{k}_{+1}-k_{{\rm in},x}}\, \bar{C}_p(\bar{k}_{+1}; x), \quad \text{(S16)}$$

where

$$C_p(k_{-1}; x) = \frac{R_{p,-1}(k, \omega_{+1}; x)}{R_{p,0}(k, \omega_{+1}; x) - 1}\; [R_{p,0}(k_{{\rm in},x}, \omega_{\rm in}; x) - 1],$$

$$\overline{C}_p(\bar{k}_{+1}; x) = \frac{R_{p,+1}(\bar{k}, \omega_{\rm in}; x)}{R_{p,0}(\bar{k}, \omega_{\rm in}; x) - 1}\; [R_{p,0}(-\bar{k}_{+1}, \omega_{+1}; x) - 1]. \quad \text{(S17)}$$

In Eq. (S15) $\boldsymbol{B}_p(k''_{x,-1}) = \hat{\boldsymbol{e}}^+_p(k''_{x,-1}) \odot \widetilde{\boldsymbol{F}}_p(k''_{x,-1})$, where $\widetilde{\boldsymbol{F}}_p(k''_{x,-1})$ is given by Eqs. (S16, S17) with the replacements $k'_{x,-1} \to k''_{x,-1}$ and $\hat{\boldsymbol{e}}^-_p(\bar{k}_{+1}) \to \hat{\boldsymbol{e}}^-_s(\bar{k}_{+1})$, and arises from the magnetic field. We note that $\overline{\boldsymbol{S}}_p(x, z)$ has components only along x and z. We recall that momenta for which $k'_{x,-1} = k'_x - \varphi'(x)$ and $k''_{x,-1} = k''_x - \varphi'(x)$ satisfy $0 \le |k'_{x,-1}|, |k''_{x,-1}| < \omega_{\rm in}/c$ give propagative modes and contribute to far-field emission, while when either $|k'_{x,-1}| > \omega_{\rm in}/c$ or $|k''_{x,-1}| > \omega_{\rm in}/c$ (evanescent modes in the STMM-vacuum interface) they contribute to near-field emission.

In order to determine the main directions into which energy is radiated from every point *x* along the STMM, and to compare with the experimental far-field measurements, we study in more detail the structure in momentum-space of the radiative part of the Poynting vector, more specifically its horizontal component at $z = 0, \bar{S}_{p,x}^{\text{rad}}(k_x', k_x''; x, 0)$. Information about the main directions of emission can be obtained by performing a partial integration of $\bar{S}_{p,x}^{\text{rad}}(k_x', k_x''; x, 0)$ over either k_x' or k_x''. Without loss of generality, we integrate over k_x'' and define a k_x' -spectrum of radiated power along the horizontal direction as follows:

$$\bar{S}_{p,x}^{\text{rad}}(k_x'; x, 0) = \int_{-\omega_{\text{in}}/c}^{\omega_{\text{in}}/c} \frac{dk_{x,-1}''}{2\pi} \bar{S}_{p,x}^{\text{rad}}(k_x', k_x''; x, 0)$$

$$= \frac{(E^{\text{in}})^2}{2\, Z_{\text{v}}} \frac{\text{sg}(k_{x,-1}')|k_{x,-1}'|}{\omega_{\text{in}}/c} \text{Re}\left\{\tilde{F}_p(k_{x,-1}')\, e^{i\, x\, k_{x,-1}'} \int_{-\omega_{\text{in}}/c}^{\omega_{\text{in}}/c} \frac{dk_{x,-1}''}{2\pi} \tilde{F}_p^*(k_{x,-1}'')\, e^{-i\, x\, k_{x,-1}''}\right\}, \quad \text{(S18)}$$

with $0 \leq |k_{x,-1}'| < \omega_{\text{in}}/c$ for propagative modes. Here, $\tilde{F}_p(k_{x,-1}')$ is a scalar given by Eq. (S16) once the unit vector is omitted. At $k_{x,-1}' = 0$ the spectrum vanishes, which means that energy is emitted vertically and one should look at the *z*-component of the radiative Poynting vector spectrum, $\bar{S}_{p,x}^{\text{rad}}(k_x'; x, 0)$, which has the same form as Eq. (S18) with $\text{sg}(k_{x,-1}')|k_{x,-1}'|$ replaced by $k_{z\text{v},-1}'$. Although both spectra can have positive and negative values, it is clear that after integration over $k_{x,-1}'$ we should have $\bar{S}_{p,z}^{\text{rad}}(x, 0) > 0$, while $\bar{S}_{p,x}^{\text{rad}}(x, 0)$ can be positive or negative depending on the dominant direction of emission (upper-right or upper-left). We can find the main directions of radiative emission by determining which $k_{x,-1}'$ most contribute to $\bar{S}_{p,x}^{\text{rad}}(k_x'; x, 0)$. The integral in the second line of Eq. (S18) gives a complex number that depends on *x* but is independent of $k_{x,-1}'$, so it is not important for determining the main contributions to the spectrum. The latter is basically the sum of several terms containing the oscillatory functions $|k_{x,-1}'|\cos(xk_{x,-1}')$ and $|k_{x,-1}'|\sin(xk_{x,-1}')$, each of them modulated by the real or the imaginary parts of $\tilde{F}_p(k_{x,-1}')$. The

interplay between them determines the main $k'_{x,-1} = k'_{x,-1}(x)$ contributions to the spectrum. To the best of our knowledge, no simple analytical form exists for $k'_{x,-1}$ for generic phase distributions.

Nonreciprocal beam steering with finite-sized STMMs: We consider the linear phase distribution $\varphi^{\text{steer}}(x) = \beta x$ and analyze the structure of the corresponding $\tilde{F}_p(k'_{x,-1})$. The real and imaginary parts of the factor $C_p(k'_{-1}; x) \sim 1/A_{p,-1}$ in Eqs. (S16, S17) have extrema at $k'_{x,-1} = \pm\omega_{\text{in}}/c$ because $|A_{p,-1}|$ presents a minimum (see Eqs. (S8,S9)). The $\bar{k}$-integral in Eq. (S16) also depends on $k'_{x,-1}$ through the Fourier transform $\text{FT}\big[\tilde{\theta}(u)\, e^{-i\,\beta u}\big]_{k'_{x,-1}+\bar{k}_{+1}}$, which is a sinc function with a single major peak at $k'_{x,-1} + \bar{k}_{+1} = -\beta$. The other factors within the $\bar{k}$-integral are $\text{FT}\big[\tilde{\theta}(u)\, e^{+i\,\beta u}\big]_{\bar{k}_{+1}}$ that has a single major peak at $\bar{k}_{+1} = \beta$ (normal incidence in forward scattering is assumed), and $\bar{C}_p(\bar{k}_{+1}; x) \sim 1/A_{p,+1}$ with extrema at $\bar{k}_{+1} = \pm\omega_{\text{in}}/c$. Therefore, $\tilde{F}_p(k'_{x,-1})$ has extrema for $k'_{x,-1} = \pm\omega_{\text{in}}/c$, $k'_{x,-1} = \mp\omega_{\text{in}}/c - \beta$, and $k'_{x,-1} = -2\beta$. The first solutions give grazing emission but is suppressed by the sinc functions of the FT's. The second solution can be discarded because they do not result in propagative reverse waves (*e.g.*, if $\beta > 0$ in forward scattering, then $k'_{x,-1} = -\omega_{\text{in}}/c - \beta$ is evanescent and $k'_{x,-1} = +\omega_{\text{in}}/c - \beta$ gives right-moving modes). Therefore, only $k'_{x,-1} = -2\beta$ gives a relevant feature and $\tilde{F}_p(k'_{x,-1}) \sim \text{sinc}[(L_x/2)(k'_{x,-1} + 2\beta)]$. Then, $\bar{S}^{\text{rad}}_{p,x}(k'_x; x, 0)$ is proportional to a sum of several terms containing the combinations $|k'_{x,-1}|\cos(xk'_{x,-1})\text{sinc}[(L_x/2)(k'_{x,-1} + 2\beta)]$ and $|k'_{x,-1}|\sin(xk'_{x,-1})\text{sinc}[(L_x/2)(k'_{x,-1} + 2\beta)]$. For any given position x on the metasurface, the numerically computed spectrum (not shown) depicts a single major peak at $k'_{x,-1}$ close to -2β that determines the main direction of emission. This peak overwhelms the peaks and dips

stemming from the factors $|k'_{x,-1}|\cos(xk'_{x,-1})$ and $|k'_{x,-1}|\sin(xk'_{x,-1})$ because the tails of the sinc function strongly suppress them. In the limit $\beta L_x \gg 1$, $k'_{x,-1} \to -2\beta$ independently of the value of *x,* which is precisely the result obtained for infinite-sized STMMs. Finally, we mention that when $|\beta| > \omega_{\text{in}}/c$ in forward scattering, we enter the regime of extreme nonreciprocity in reverse scattering. There is no radiative emission (Figs. 3f,h) and surface waves are launched on the metasurface.

Nonreciprocal focusing with finite-sized STMMs: Let us now tackle the focusing phase distribution $\varphi^{\text{focus}}(x) = -(\omega_{+1}/c)\{[(x - x_f)^2 + z_f^2]^{1/2} - z_f\}$ (we assume normal incidence in forward scattering). We consider in the following the off-axis nonreciprocal focusing situation corresponding to the experiment of Figs. 4c,d of the main text. Supplementary Figs. 7a,b show the integrands $\bar{S}_{p,x}(k'_x, k''_x; x, z)$ and $\bar{S}_{p,z}(k'_x, k''_x; x, z)$ at the right edge of the STMM, $(x, z) = (L_x/2{,}0)$. A complex structure of peaks and troughs is observed, with the largest ones lying within the evanescent sector, indicating the excitation of surface modes and the generation of near-field emission from the finite-sized STMM in the reverse scattering process. Smaller structures within the propagative sector contribute to far-field radiation. In contrast to beam steering, there is no threshold to excite surface waves in reverse scattering, and extreme nonreciprocity shows a co-existence of strong surface modes and weak radiative modes. Integration over k'_x and k''_x in the radiative sector $|k'_{x,-1}|, |k''_{x,-1}| < \omega_{\text{in}}/c$ results in partial cancellation between peaks and troughs, and gives net radiated power components $\bar{S}_{p,x}^{\text{rad}}(L_x/2{,}0) > 0$ and $\bar{S}_{p,z}^{\text{rad}}(L_x/2{,}0) > 0$, *i.e.*, far-field emission along positive *x*- and *z*-directions. Right-propagating modes $0 \le k'_{x,-1}, k''_{x,-1} < \omega_{\text{in}}/c$ are the relevant ones for far-field emission from the right edge of the metasurface. Supplementary Figs. 7d,e show the corresponding integrands at the left edge of the STMM, $(x, z) = (-L_x/2{,}0)$.

Again, most of the structures lie in the evanescent sector, and the net radiative contributions give $\bar{S}_{p,x}^{\text{rad}}(-L_x/2,0) < 0$ and $\bar{S}_{p,z}^{\text{rad}}(-L_x/2,0) > 0$. Left-propagating modes $-\omega_{\text{in}}/c < k'_{x,-1}, k''_{x,-1} \leq 0$ are the ones that matter for far-field emission from the left edge of the metasurface.

Next, we study the radiative power spectrum at different points on the metasurface, and start by considering the right-edge of the STMM. Supplementary Fig. 7c depicts $\bar{S}_{p,x}^{\text{rad}}(k'_x; L_x/2,0)$, for right-propagating modes. showing the existence of four peaks. The first one at $(\omega_{\text{in}}/c)^{-1}k'_{x,-1} = 1$ gives grazing propagation to the right and is not measured by the detector in our experiments because the maximum scanning angles are $\approx \pm 75^{\text{o}}$. The next peak, whose emitted radiative signal is denoted as ER1, is located at $k'^{\text{ER1}}_{x,-1} \approx 0.78 \times \omega_{\text{in}}/c$. The angle between the direction of emission and the plane of the metasurface is $g_{\text{ER1}} = \arccos[k'^{\text{ER1}}_{x,-1}(\omega_{\text{in}}/c)^{-1}] \approx 39^{\text{o}}$, indicating that ER1 contributes to the highest intensity signal in the right-side of Fig. 4c. The emission signal ER2 at $k'^{\text{ER2}}_{x,-1} \approx 0.55 \times \omega_{\text{in}}/c$ makes an angle $g_{\text{ER2}} = \arccos[k'^{\text{ER2}}_{x,-1}(\omega_{\text{in}}/c)^{-1}] \approx 57^{\text{o}}$ and contributes to the lower-intensity peak in the right-side of Fig. 4c. Finally, the peak at $k'_{x,-1} \approx 0.32 \times \omega_{\text{in}}/c$ results in a low-power signal below the experimental resolution. Just as for $x = L_x/2$, points x on the STMM such that $x_f < x \leq L_x/2$ also result in $\bar{S}_{p,x}^{\text{rad}}(x,0) > 0$ and contribute to the far-field measured signal in the right side of Fig. 4c. Their radiated power falls around the signals E1R and E2R radiated from $x = L_x/2$. The spectrum $\bar{S}_{p,x}^{\text{rad}}(k'_x; -L_x/2,0)$ for radiative emission from the left-edge of the STMM is shown in Supplementary Fig. 7f for left-propagating modes, which indicates the presence of three troughs (for emission towards the left we need to look for troughs rather than peaks because $\bar{S}_{p,x}^{\text{rad}}$ must be negative). The first one at $k'^{\text{EL}}_{x,-1} \approx -0.3 \times \omega_{\text{in}}/c$ corresponds to emission towards the left that we denote as EL, makes an angle $g_{\text{EL}} = \arccos[-k'^{\text{EL}}_{x,-1}(\omega_{\text{in}}/c)^{-1}] \approx 72^{\text{o}}$ between the direction of propagation and the

metasurface's plane, and contributes to the far-field signal on the left-side of Fig. 4c. The trough at $k'_{x,-1} \approx -0.6 \times \omega_{\rm in}/c$ emits a signal that is below the experimental resolution, and the one at $k'_{x,-1} \approx -0.9 \times \omega_{\rm in}/c$ results in almost grazing radiation to the left. Points x on the STMM such that $-L_x/2 \leq x < x_f$ also result in $\bar{S}^{\rm rad}_{p,x}(x,0) < 0$ and contribute to the far-field measured signal on the left-side of Fig. 4c.

Supplementary Fig. 7g indicates the geometry of the STMM and the scanning arc made by the detector in the far-field. Our goal now is to relate the angles of main emission from the right ($g_{\rm ER1}, g_{\rm ER2}$) and left ($g_{\rm EL}$) edges of the metasurface, with the angles α at the crossings between those emission directions and the measurement arc (these angles are measured from the normal at the STMM's center, $\alpha > 0$ for ER1 and ER2, and $\alpha < 0$ to EL. One can easily derive the identity $(L_x/2d)\tan(g) + \cos(\alpha) = \tan(g)\sin(\alpha)$, where d is the distance between the detector and the center of the STMM. The solutions to this equation are

$$\alpha = \arccos\left\{\frac{-\left(\frac{L_x}{2d}\right)\tan(g) + \sqrt{\tan^2(g) + [1-(L_x/2d)^2]\tan^4(g)}}{1+\tan^2(g)}\right\}. \tag{S19}$$

Therefore, the angles that the signal from ER1, ER2, and EL make with the normal to the STMM are $\alpha_{\rm ER1} \approx +54^{\rm o}$, $\alpha_{\rm ER2} \approx +37^{\rm o}$, and $\alpha_{\rm EL} \approx -22^{\rm o}$, respectively. These values are in very good agreement with the measured values of the features in Figs. 4c,d. The strengths of the signals emitted from the right of the STMM are stronger than those emitted from the left, and hence the peak at $\alpha_{\rm ER1}$ is higher than the one at $\alpha_{\rm EL}$.

Analytical considerations for the radiative power spectrum in focusing: In this sub-section we provide analytical expressions that approximate the numerically calculated locations $k'_{x,-1}(x)$ of the peaks and troughs in Supplementary Figs. 7c,f. As already mentioned, the spectrum

$\bar{S}_{p,x}^{\text{rad}}(k_x'; x, 0)$ is basically the sum of several terms containing the oscillatory functions $|k_{x,-1}'|\cos(xk_{x,-1}')$ and $|k_{x,-1}'|\sin(xk_{x,-1}')$, each of them modulated by the real or the imaginary parts of $\tilde{F}_p(k_{x,-1}')$. In focusing, $\text{Re}\tilde{F}_p\big(k_{x,-1}'\big)$ and $\text{Im}\tilde{F}_p(k_{x,-1}')$ have several extrema arising from the different factors in Eq. (S16), in particular from the Fourier transform $\text{FT}\left[\tilde{\theta}(u)\, e^{-i\varphi^{\text{focus}}(u)}\right]_{k_{x,-1}'+\bar{k}_{+1}}$ (see Supplementary Note 4). Those extrema take place at $k_{x,-1}' = \pm\omega_{\text{in}}/c$, $k_{x,-1}' = \mp\omega_{\text{in}}/c\ + \kappa_{R/I,j^-}^-$, and $k_{x,-1}' = \kappa_{R/I,j^-}^- - \kappa_{R/I,j^+}^+$. The structure of $\bar{S}_{p,x}^{\text{rad}}(k_x'; x, 0)$ is the result of a complex interplay between the several factors appearing in Eqs. (S16) and (S18). A general formula that accounts for all the features present in $\bar{S}_{p,x}^{\text{rad}}(k_x'; x, 0)$ at every point x on the STMM is hard to find. However, one can notice that the separation between consecutive peaks for $k_{x,-1}' > 0$ in Supplementary Fig. 7c is approximately constant (but not for the troughs), and the same holds true for the separation between consecutive troughs for $k_{x,-1}' < 0$ in Supplementary Fig. 7f (but not for the peaks). This observation will enable us to propose simple functions that successfully approximate the locations of the $k_{x,-1}' > 0$ peaks for $x > x_f$ (as in Supplementary Fig. 7c), and the locations of the $k_{x,-1}' < 0$ troughs for $x < x_f$ (as in Supplementary Figs. 7f).

We first consider points on the STMM to the right of the focal point, $x > x_f$, that have negative phase gradient $\varphi'^{\text{focus}}(x) < 0$. In this situation the total radiative power $\bar{S}_{p,x}^{\text{rad}}(x, 0)$ is positive and, therefore, $k_{x,-1}' > 0$ modes where $\bar{S}_{p,x}^{\text{rad}}(k_x'; x, 0)$ is positive and maximum are the relevant ones for determining the emission directions, *i.e.*, positive peaks in the spectrum. Serendipitously, we found that for $x = L_x/2$ the function $f_1(m_1) = \omega_{\text{in}}/c\ + m_1\varphi'^{\text{focus}}(L_x/2)$ (m_1 a positive integer) gives a very good approximation for the locations of the $k_{x,-1}' > 0$ peaks depicted in the numerically-evaluated spectrum of Fig. 7c. Indeed, from the figure we get $k_{x,-1}' \approx (0.78, 0.55, 0.32) \times \omega_{\text{in}}/c$, respectively for the right, central, and left peaks. Using that

$\varphi'^{\text{focus}}(L_x/2) = -0.2272 \times \omega_{\text{in}}/c$, $f_1(m_1)$ gives $k'_{x,-1} = (0.7728, 0.5456, 0.3184) \times \omega_{\text{in}}/c$ for $m_1 = 1,2,3$ (there is another very small peak for $m_1 = 4$ at $k'_{x,-1} = 0.0912 \times \omega_{\text{in}}/c$ that is barely seen in the figure). The angle between the STMM plane and the propagation direction of the strongest signal ER1 emitted from $L_x/2$, that is $g_{\text{ER1}} \approx 39^{\text{o}}$, is well approximated by

$$g_{+}^{\text{th}} = \arccos[1 + (\omega_{\text{in}}/c\,)^{-1}\varphi'^{\text{focus}}(L_x/2)] = 39.39^{\text{o}}, \quad \text{(S20)}$$

and the angle for the second-strongest peak, i.e. $g_{\text{ER2}} \approx 57^{\text{o}}$, is also well approximated by $g_{\text{ER2}}^{\text{th}} = \arccos[1 + (\omega_{\text{in}}/c\,)^{-1} 2\, \varphi'^{\text{focus}}(L_x/2)] = 58.93^{\text{o}}$. These values result in predicted scanning angles for ER1 of $\alpha_{\text{R}}^{\text{th}} = +53.27^{\text{o}}$ and for ER2 of $\alpha_{\text{ER2}}^{\text{th}} = +34.66^{\text{o}}$. A similar analysis can be done for points on the STMM to the left of the focal point, $x < x_f$, whose phase gradient is positive. In this situation $\overline{S}_{p,x}^{\text{rad}} < 0$, and then $k'_{x,-1} < 0$ modes where $\overline{S}_{p,x}^{\text{rad}}(k'_x; x, 0)$ is as negative as possible are the relevant ones, *i.e.*, we need to look at the negative troughs. We found that the function $f_2(m_2) = m_2[-\omega_{\text{in}}/c + \varphi'^{\text{focus}}(-L_x/2)]$ (m_2 a positive integer) gives a good approximation to the locations of $k'_{x,-1} < 0$ troughs for the left-edge of the STMM. Supplementary Fig. 7f shows three troughs at $k'_{x,-1} \approx -(0.30, 0.58, 0.88) \times \omega_{\text{in}}/c$. The function $f_2(m_2)$ gives $k'_{x,-1} = -(0.2814, 0.5836, 0.8442) \times \omega_{\text{in}}/c$ for $m_2 = 1,2,3$, where we used that $\varphi'^{\text{focus}}(-L_x/2) = 0.7186 \times \omega_{\text{in}}/c$. The angle for the EL signal from $-L_x/2$, that is $g_{\text{EL}} \approx 72^{\text{o}}$, is well approximated by

$$g_{-}^{\text{th}} = \arccos[1 - (\omega_{\text{in}}/c)^{-1}\varphi'^{\text{focus}}(-L_x/2)] = 73.66^{\text{o}}, \quad \text{(S21)}$$

which results in a predicted scanning angle for EL of $\alpha_{\text{L}}^{\text{th}} = -20.36^{\text{o}}$.

Supplementary Note 8: Extreme Nonreciprocity in STMMs with Arbitrary Nonlinear Phase Distributions. When nonlinear phase distributions $\varphi_{\text{NL}}(\boldsymbol{r})$ different from the focusing one are

dynamically imprinted on the STMM, extreme nonreciprocity still occurs, with the scattered field in the reverse process containing low-power propagative waves and evanescent surface modes. For the infinite-sized STMM and the scattering process $(-\boldsymbol{k}_{\text{in}} - \nabla\varphi_{\text{NL}}, \omega_{\text{in}} + \Omega) \rightarrow (-\boldsymbol{k}_{\text{in}} - 2\,\nabla\varphi_{\text{NL}}, \omega_{\text{in}})$ in the integrand of Eq. (S12), the boundary between the propagative and evanescent sectors is given by $(\omega_{\text{in}}/c)^2 - |\boldsymbol{k}_{\text{in}} + 2\,\nabla\varphi_{\text{NL}}(\boldsymbol{r})|^2 = 0$. Regions of the STMM where the phase gradient makes the left-hand-side of the previous relation positive will radiate waves into the far-field, while regions where it makes it negative correspond to evanescent surface waves. Scattered power spatial distributions more complex than that shown in Fig. 4b are possible, presenting multiple alternating propagative and evanescent sectors. For example, engineering a 5th-order polynomial nonlinear phase distribution such that its gradient is given by the "Mexican hat" form $\nabla\varphi_{\text{NL}}^{\text{Mh}}(\boldsymbol{r}) = \hat{\boldsymbol{x}}\,(x^2 - \lambda_{\text{in}}^2)^2/\lambda_{\text{in}}^5$, the corresponding structure to Fig. 4b has three evanescent sectors (given by $x < -\tilde{x}_+$, $-\tilde{x}_- < x < \tilde{x}_-$, and $x > \tilde{x}_+$) separated by two propagative "ribbons" (located at $-\tilde{x}_+ < x < -\tilde{x}_-$ and $\tilde{x}_- < x < \tilde{x}_+$). Here, $\tilde{x}_\pm = \lambda_{\text{in}}\sqrt{1 \pm \sqrt{\pi}}$ and we assume normal incidence ($\boldsymbol{k}_{\text{in}} = \boldsymbol{0}$) for the forward input. On the other hand, when the STMM has finite size, the power profile emitted into the near- and far-field zones can be computed following the same procedure described in Supplementary Note 7. The resulting far-field power profile can have more features than the one found for focusing, *e.g.*, a "multi-wedge" structure with several distinguishable peaks.

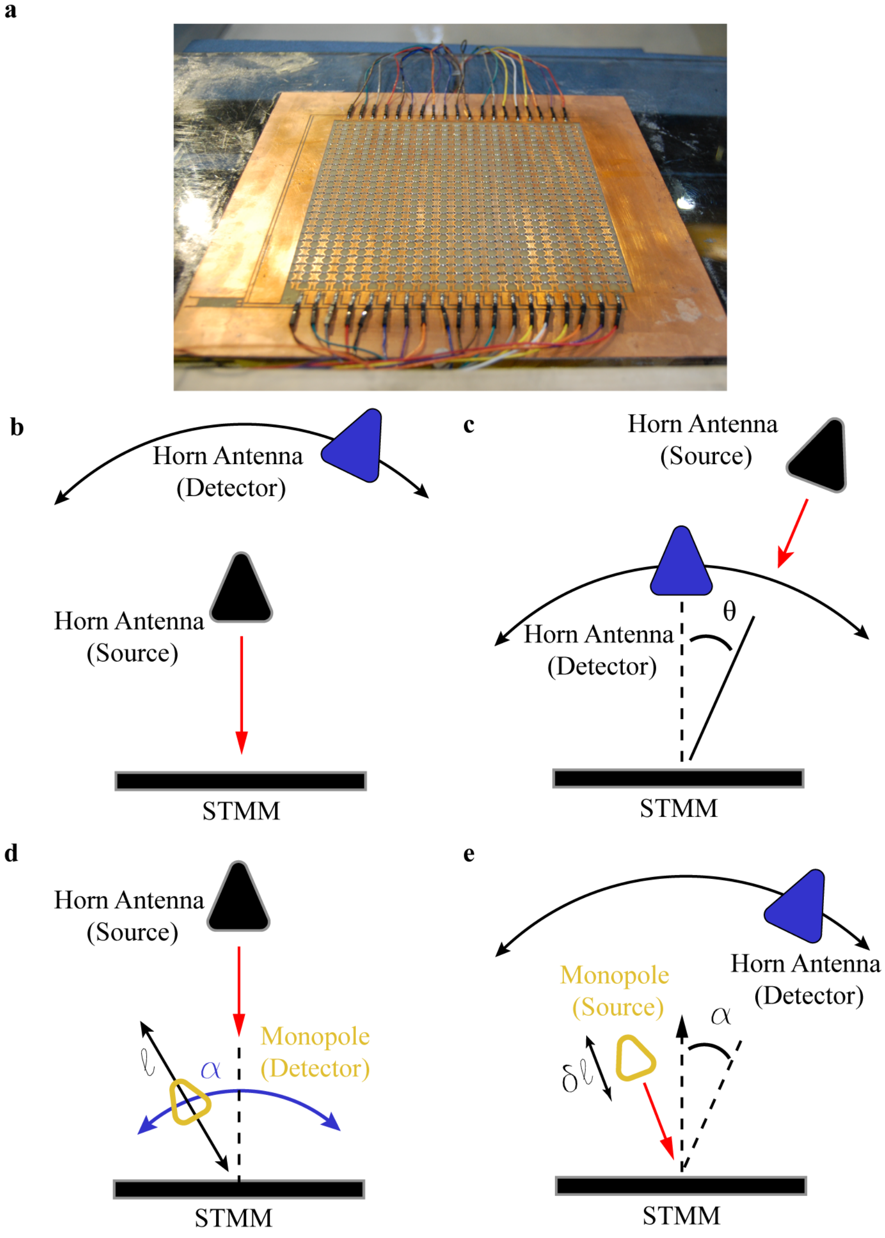

Supplementary Figure 1 | a, Photograph of our STMM. **b,** Schematic of the experimental set-up for dynamical beam steering experiments. **c,** Schematic for reverse nonreciprocity beam steering experiments. **d,** Same as **b** for dynamical focusing experiments. **e,** Same as **c** for nonreciprocal focusing experiments.

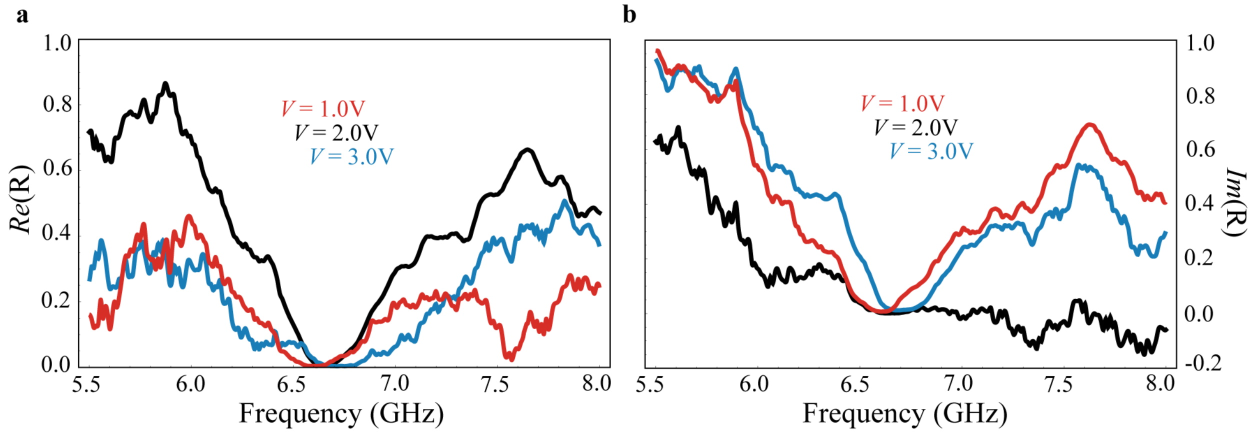

Supplementary Figure 2 | a, Measured real part of the reflectivity as a function of frequency for the unmodulated metasurface with all varactors uniformly biased with voltages $V = 1$ V (red), $V = 2$ V (black), and $V = 3$ V (blue). **b**, Same for the imaginary part. Measurements are performed with *p*-polarized light at 10° angle of incidence.

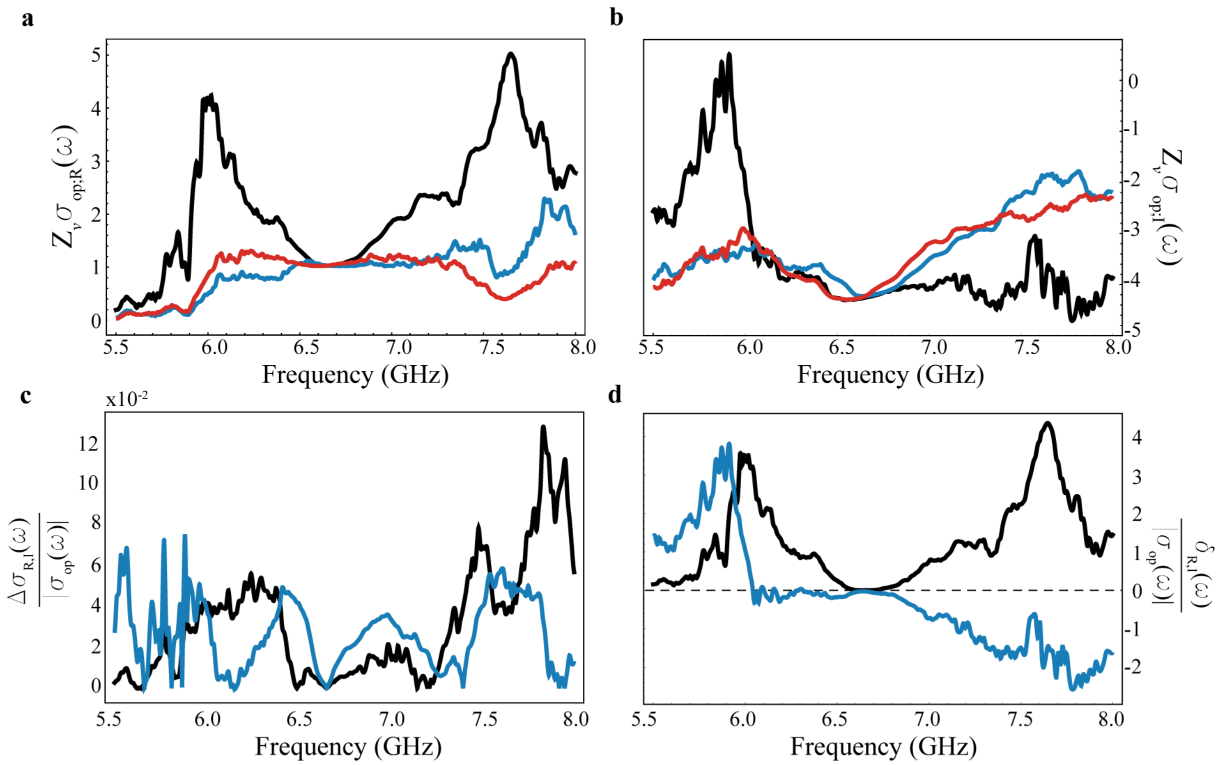

Supplementary Figure 3 | a, Extracted real part of the unmodulated conductivity as a function of frequency for the various bias voltages V = 1 V (red), V =2 V (black), and V = 3 V (blue). Note that intrinsic resonances associated to the unit cell design appear around 6.0 GHz and 7.6 GHz for V =2 V. **b**, Same for the imaginary part. **c,** Estimated real (black) and imaginary (blue) parts of the conductivity modulation $\Delta\sigma(\omega)$ as a function of frequency for the operating voltage V_{op} = 2 V. **d,** Real (black) and imaginary (blue) parts of the difference $\delta(\omega) \equiv \sigma_{\text{op}}(\omega) - [\sigma^{V=3\text{V}}(\omega) + \sigma^{V=1\text{V}}(\omega)]/2$.

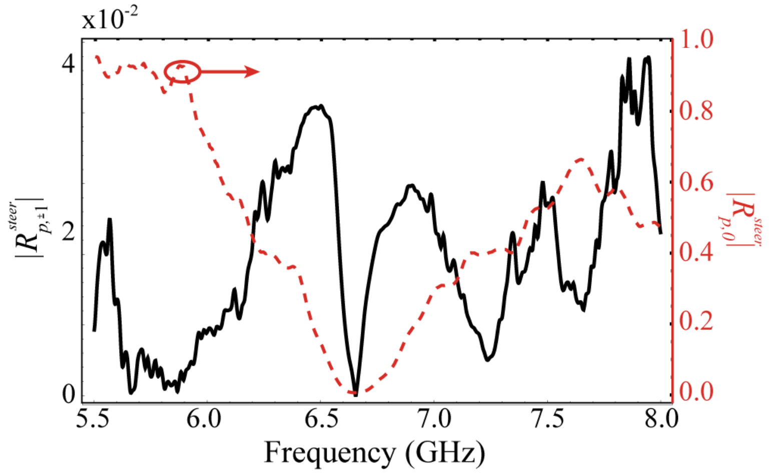

Supplementary Figure 4 | Computed p-polarization modulus of the reflectivity $R^{\text{steer}}_{p,n}(k_{\text{x,in}}, \omega; x)$ for $n = 0$ (red) and $n = \pm 1$ (black) harmonics in beam steering as a function of the incoming frequency. We use a normally incident plane wave, a phase gradient β_x =44 m^{-1}, and modulation frequency of 50 kHz. Note that a few peaks appear in the conversion efficiency to the ± 1 harmonics, and in the main paper we decided to operate around the peak at 6.9 GHz.

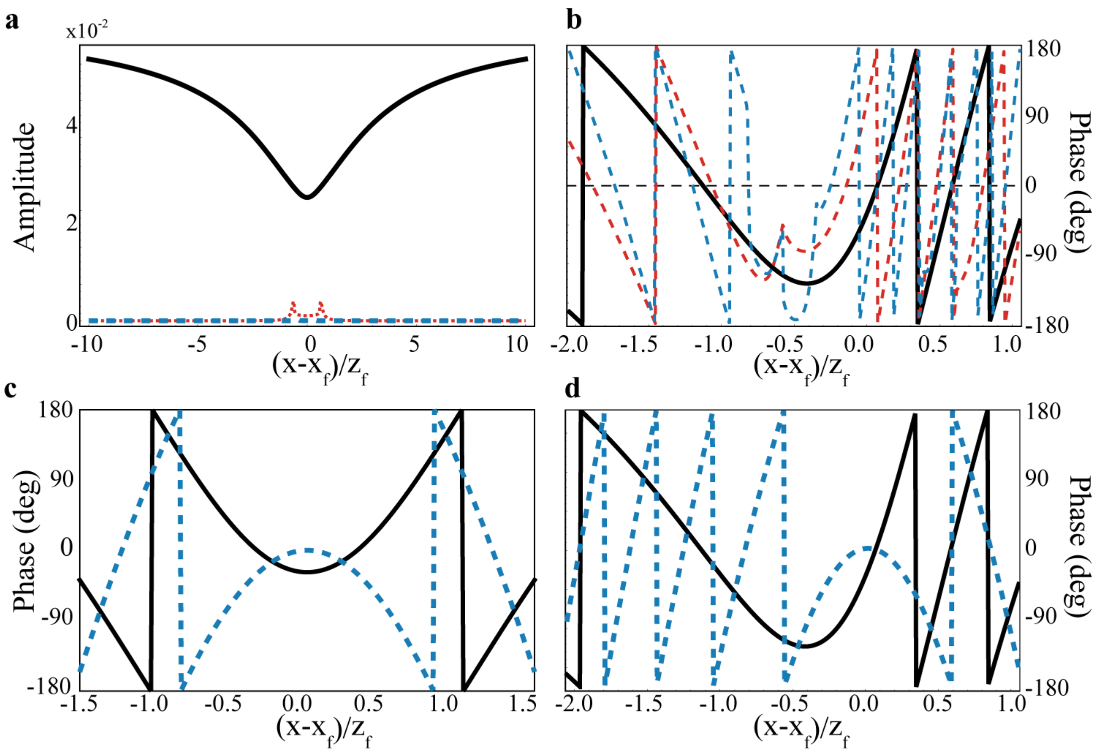

Supplementary Figure 5 | a, Calculated p-polarization amplitude of the focusing reflection coefficients $R^{\text{focus}}_{p,\bar{n}=+1,n}(k_{\text{in},x},\omega_{\text{in}};x)$ as a function of position, for the n = +1 (black), +2 (dashed red), and +3 (dashed blue) harmonics and focusing phase distribution with $x_f = 6$ cm , $z_f = 15$ cm. **b,** Same for the phase. **c,** Comparison of the phase of the reflection coefficient $R^{\text{focus}}_{p,\bar{n}=+1,n=+1}(k_{\text{in},x},\omega_{\text{in}};x)$ (black) with $\varphi^{\text{focus}}_{\bar{n}=+1}(x)$ (blue) as a function of position for on-axis focusing, $x_f = 0$ cm , $z_f = 15$ cm. **d,** Same as **c** for off-axis focusing, $x_f = 6$ cm , $z_f = 15$ cm. In all plots we assume a 6.9 GHz normally incident plane wave and modulation frequency of 50 kHz.

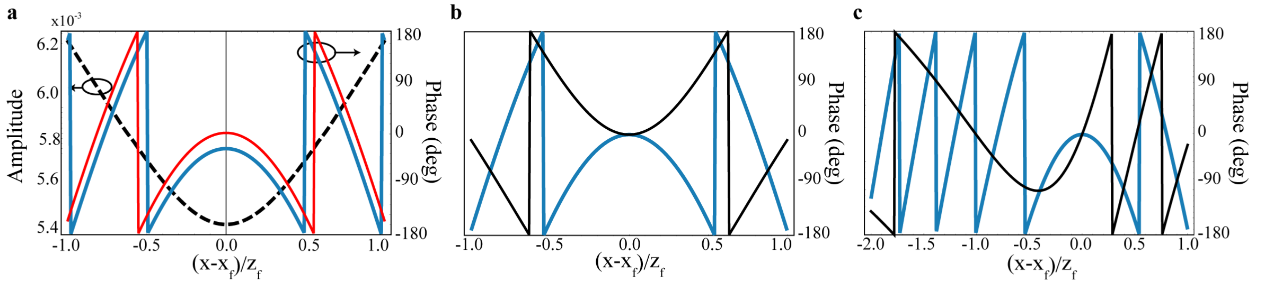

Supplementary Figure 6 | a, Amplitude (dashed black) and phase (blue) of the total reflection coefficient $R_{p,\bar{n},n}^{\text{tot,focus}}(k_{\text{in},x}, \omega_{\text{in}}; x)$ as a function of position for $n = \bar{n} = +1$ and $x_f = 6$ cm , $z_f = 15$ cm. The phase of the focusing profile is shown in red. **b,** Comparison of the phase of the reflection coefficient $R_{p,\bar{n}=+1,n=+1}^{\text{focus}}(k_{\text{in},x}, \omega_{\text{in}}; x)$ (black) with that of $R_{p,\bar{n}=+1,n=+1}^{\text{tot,focus}}(k_{\text{in},x}, \omega_{\text{in}}; x)$ (blue) for on-axis focusing, $x_f = 0$, $z_f = 15$ cm. **c,** Idem for off-axis focusing, $x_f = 6$ cm , $z_f = 15$ cm. In all plots we assume a 6.9 GHz normally incident plane wave and modulation frequency of 50 kHz.

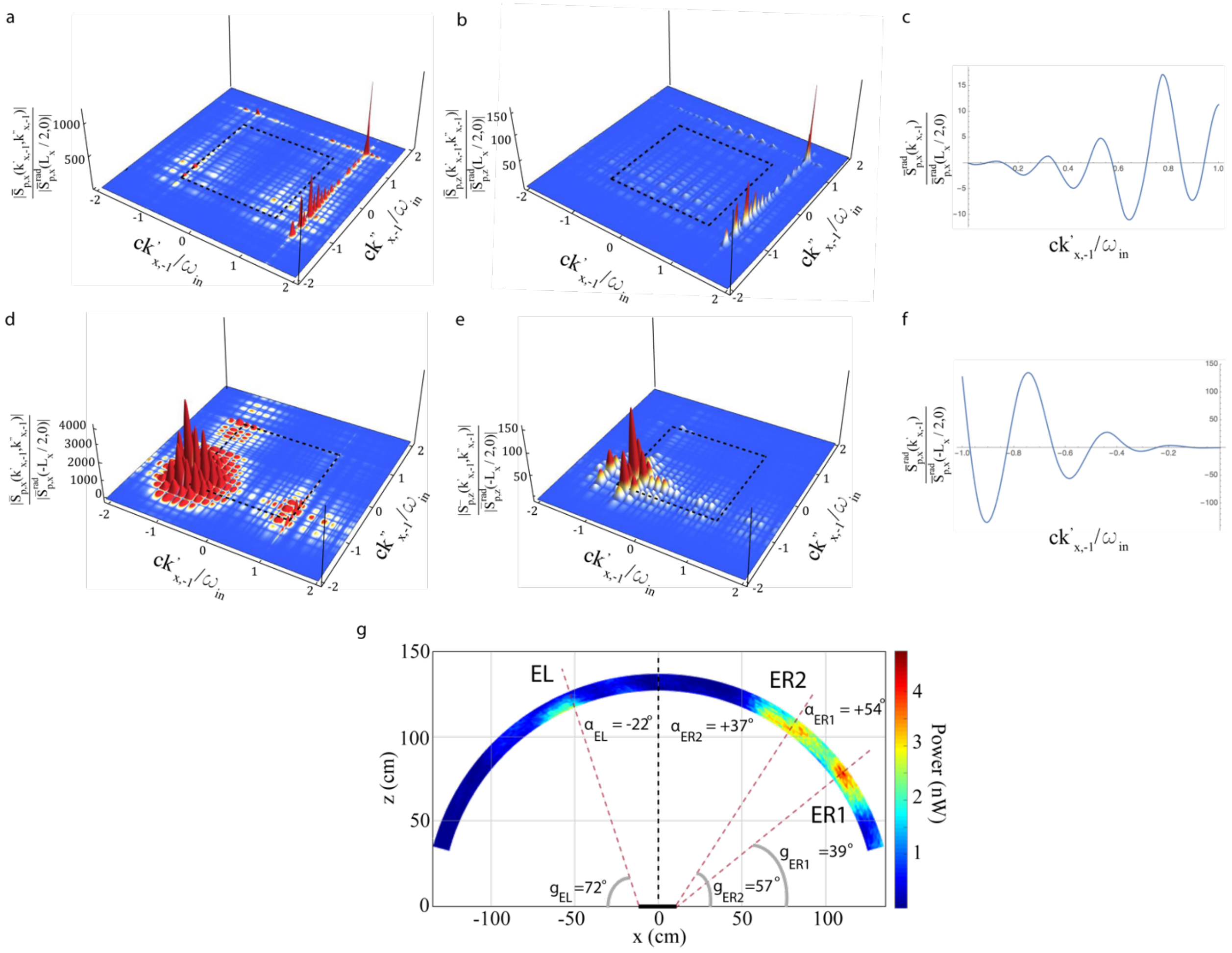

Supplementary Figure 7 | a-c, Poynting vector at $(L_x/2{,}0)$: **a,** $|\bar{S}_{p,x}(k'_{x,-1}, k''_{x,-1})|$ in the $(k'_{x,-1}, k''_{x,-1})$ plane, normalized to $|\bar{S}^{\rm rad}_{p,x}(L_x/2{,}0)|$. **b,** $|\bar{S}_{p,z}(k'_{x,-1}, k''_{x,-1})|$ normalized to $\bar{S}^{\rm rad}_{p,z}(L_x/2{,}0)$. In both cases, the region inside (outside) the dashed square corresponds to propagative (evanescent) modes. **c,** $\bar{S}^{\rm rad}_{p,x}(k'_{x,-1})$ shown for right-propagating modes, normalized to $\bar{S}^{\rm rad}_{p,x}(L_x/2{,}0)$. **d-f,** Poynting vector at $(-L_x/2{,}0)$: **d,** $|\bar{S}_{p,x}(k'_{x,-1}, k''_{x,-1})|$ normalized to $|\bar{S}^{\rm rad}_{p,x}(-L_x/2{,}0)|$. **e,** $|\bar{S}_{p,z}(k'_{x,-1}, k''_{x,-1})|$ normalized to $\bar{S}^{\rm rad}_{p,z}(-L_x/2{,}0)$. **f,** $\bar{S}^{\rm rad}_{p,x}(k'_{x,-1})$ shown for left-propagating modes, normalized to $|\bar{S}^{\rm rad}_{p,x}(-L_x/2{,}0)|$. **g,** Geometry showing the STMM and the scanned far-field signal. The directions of propagation for the main radiative emissions ER1 and ER2 from $(L_x/2{,}0)$, and EL from $(-L_x/2{,}0)$, are indicated. Also shown are the approximated numerically calculated angles that those emission directions form with the plane of the STMM, and the corresponding approximated numerically calculated scanning angles (measured from the normal at the STMM's center), see Eq. (S19). The corresponding calculated angles using the theory approach presented in Supplementary Note 7 are $g_+^{\rm th} \equiv g_{\rm ER1} = 39.39^{\rm o}$, $g_{\rm ER2} = 58.93^{\rm o}$, $g_-^{\rm th} \equiv g_{\rm EL} = 73.66^{\rm o}$, and $\alpha_{\rm R}^{\rm th} \equiv \alpha_{\rm ER1} = +53.27^{\rm o}$, $\alpha_{\rm ER2} = +34.66^{\rm o}$, $\alpha_{\rm L}^{\rm th} \equiv \alpha_{\rm EL} = -20.36^{\rm o}$. In all panels we use the experimental parameters $n = \bar{n} = +1$, $x_f = 6$ cm, $z_f = 15$ cm, $L_x = 19$ cm, $d = 130$ cm, $\omega_{\rm in} = 2\pi \times 6.9$ GHz, and $\Omega = 2\pi \times 50$ kHz.
